# Two types of C-terminal regions of RNA-binding proteins play distinct roles in stress tolerance of *Synechocystis* sp. PCC 6803

**DOI:** 10.1093/femsle/fnac021

**Published:** 2022-02-25

**Authors:** Yueming Zhang, Dongqing Wu, Yali Wang, Xudong Xu

**Affiliations:** The State Key Laboratory of Freshwater Ecology and Biotechnology, Institute of Hydrobiology, Chinese Academy of Sciences, Wuhan, Hubei 430072, China; University of Chinese Academy of Sciences, Beijing 100049, China; The State Key Laboratory of Freshwater Ecology and Biotechnology, Institute of Hydrobiology, Chinese Academy of Sciences, Wuhan, Hubei 430072, China; University of Chinese Academy of Sciences, Beijing 100049, China; The State Key Laboratory of Freshwater Ecology and Biotechnology, Institute of Hydrobiology, Chinese Academy of Sciences, Wuhan, Hubei 430072, China; The State Key Laboratory of Freshwater Ecology and Biotechnology, Institute of Hydrobiology, Chinese Academy of Sciences, Wuhan, Hubei 430072, China

**Keywords:** RRM-type RNA-binding protein, C-terminal region, cold adaptability, salt tolerance, cyanobacteria

## Abstract

In the phylogenetic tree of RRM-type Rbps (RNA-binding proteins) in cyanobacteria, Rbp1 of *Synechocystis* 6803, with a single RRM (RNA recognition motif) region and a C-terminal glycine-rich region, and Rbp2, without the C-terminal region, both belong to the cluster I, whereas Rbp3 with a different type of C-terminal region is in the cluster II. Rbp1 is required for the cold adaptability of the cyanobacterium, and Rbp3 is for salt tolerance. Here, we report that the C-terminal region of Rbp1 is not required for the cold adaptability function but the C-terminal region of Rbp3 can direct the RRM of Rbp1 to the salt tolerance function. Bioinformatic and experimental analyses indicate that Rbps in cyanobacteria should be classified as two types. It is the first report for the distinct roles of C-terminal regions of Rbps in stress tolerance of cyanobacteria.

## Teaser

Of the two types of C-terminal sequences of RNA-binding proteins in cyanobacteria, type I is dispensable, whereas type II can direct the function of the protein.

## Introduction

RNA-binding proteins (Rbps) are found in both prokaryotes and eukaryotes (Glisovic *et al*. 2008, Holmqvist and Vogel 2018). Their interactions with RNA sequences are based on RNA-binding domains, such as the RNA-recognition motif (RRM), the K homology domain, the Sm or Sm-like domain, but there are also proteins with RNA-binding activities but without those classic domains (Corley *et al*. 2020). Rbps are involved in regulation of various cellular processes. In bacteria, Rbps play important roles in protection and scaffolding of RNA, transcription termination, translational control, matchmaking between small regulatory RNAs and target mRNAs, recruitment of the RNA degradosome, etc (Holmqvist and Vogel 2018, Ng Kwan Lim *et al*. 2021).

Cyanobacteria are oxygenic photosynthetic bacteria, widely distributed in marine and freshwater bodies, saline lakes, and terrestrial environments (Castenholz 2001) and play important roles in the biogeochemical cycling of carbon, oxygen, and nitrogen (Flombaum *et al*. 2013, Hutchins and Fu 2017). To adapt to different environments and to respond to fluctuations of environmental factors, cyanobacteria developed molecular mechanisms to regulate gene expression at multiple levels. *Synechocystis* sp. PCC 6803 (hereafter *Synechocystis* 6803) is a unicellular mesophilic cyanobacterium, and the optimal growth temperature range is from 25°C to 40°C. In *Synechocystis* 6803, Rbps have been shown to be required for the cold acclimation, overwintering (Tan *et al*. 2011), salt tolerance (Wang and Xu 2013), and localization of certain mRNAs (Mahbub *et al*. 2020). In *Anabaena variabilis*, a filamentous heterocyst-forming cyanobacterium, *rbpA1* was shown to repress the initiation of heterocyst differentiation at a low temperature in the presence of nitrate (Sato and Wada 1996).

RRM-type Rbps in cyanobacteria consist of a single RRM region (slightly over 80 aa) and a C-terminal region (from 0 to around 130 amino acid residues, 0 means lack of the C-terminal region in some Rbps). Typically, RRM possesses a β1-α1-β2-β3-α2-β4 topology, in which two α-helices are packed on a four-stranded β-sheet; in most cases, two conserved aromatic side-chains in RNP1 (ribonucleoprotein domain 1, β3-strand) and one conserved in RNP2 (β1-strand) are involved in direct interaction with the RNA substrate (Cléry *et al*. 2008, Daubner *et al*. 2013). Based on the two clusters in the phylogenetic tree, RRM-type Rbps in cyanobacteria were divided into two classes; furthermore, class (cluster) I Rbps were divided into two types: type I, with a stretch of glycine-rich sequence at the C-terminal end of the protein and conserved boxes for cold response in the 5′ UTR of the gene; type II, lacking the C-terminal region (protein) and the conserved boxes for cold response (gene) (Maruyama *et al*. 1999, Hamano *et al*. 2004). However, the criteria for the two types of class I are questionable. For example, of the three RRM Rbps in *Synechocystis* 6803, Rbp1 (Sll0517) and Rbp2 (Ssr1480) belong to the class I, Rbp1 is a typical type I member according to the criteria, Rbp2 lacks the C-terminal glycine-rich region but possesses the conserved boxes for cold response in the 5′ UTR (see Fig. 3 in the literature by Maruyama *et al*. 1999). Therefore, the conserved boxes in 5′ UTR is not a reliable criterion for typing of class I Rbps. Furthermore, the function of the C-terminal glycine-rich region of Rbp1 has not been shown. Rbp1 and Rbp2 play very different roles in the cold adaptability of *Synechocystis* 6803. An *rbp1* deletion mutant showed a great decrease in growth rate at 15°C compared to the wild type, whereas an *rbp2* deletion mutant showed only a slight decrease (Tan *et al*. 2011). Whether the C-terminal glycine-rich region determines the functional difference between Rbp1 and Rbp2 needs to be investigated.

Unlike Rbp1 and Rbp2, Rbp3 (Slr0193) in *Synechocystis* 6803 represents class (cluster) II Rbps in the phylogenetic tree and is required for the salt tolerance of the cyanobacterium (Wang and Xu 2013). Compared to the C-terminal region (21 aa) of Rbp1, that of Rbp3 is much longer (68 aa) and not rich in glycine. The function of this type of C-terminal region has not been investigated either. In this study, we investigated the functions of the C-terminal regions of Rbp1 and Rbp3. Our results showed that it is the RRM region rather than the C-terminal glycine-rich region that makes Rbp1 greatly different from Rbp2 in the role in cold adaptability and that the C-terminal region of Rbp3 can divert the function of the Rbp1 RRM to salt tolerance. We propose that Rbps in cyanobacteria should be classified as two types according to the phylogenetic relationship and the function of C-terminal region and that type I (the former class I) should not be further divided based on the C-terminal region.

## Materials and methods

### Strains and culture conditions

*Synechocystis* sp. PCC 6803 and derivatives were grown in BG11 in flasks on a rotary shaker at 30°C under continuous illumination of 30 μE m^–2^ s^–1^. Erythromycin (5 μg mL^–1^), kanamycin (25 μg mL^–1^) or spectinomycin (10 μg mL^–1^) was added as needed. To test the growth under salt stress, cells were cultured in BG11 with 1 M NaCl without shaking.

### Construction of plasmids and mutants

Molecular manipulations were performed according to standard methods or according to manufacturers′ instructions. Clones of PCR products were confirmed by sequencing. Transformation of *Synechocystis* 6803 was performed according to the standard method (Williams 1988). Complete segregation of mutants was confirmed by PCR. The processes of plasmid construction and the characteristics of mutant strains are described in Table S1 in the supplemental material, but those plasmids for generation of *Synechocystis* mutants are briefly described here.

The plasmid pHB5509 carries the upstream-Sp^r^-downstream structure (‘upstream’ stands for the sequence upstream of the target gene; ‘downstream’ stands for the sequence downstream of the target gene) for generating the Δ*rbp3* mutant; pHB5511 carries the upstream-*rbp1*-*3*-Km^r^-downstream structure for generating the *rbp1*-*3*b mutant, in which the RRM region sequence of *rbp3* was replaced with the counterpart of *rbp1*; pHB5899 carries the upstream-*rbp3*-Km^r^-downstrem structure for generating the WT(*rbp3*-K) strain; pHB5971 carries the upstream-Km^r^-downstream structure for generating the Δ*rbp1* mutant; pHB6001 carries the upstream-*rbp1*(delC)-Km^r^-downstream structure; pHB6003 carries the upstream-*rbp1*-*3*-Km^r^-downstream structure for generating the *rbp1*-*3*a mutant, in which the C-terminal region sequence of *rbp1* was replaced with the longer C-terminal region of *rbp3*; pHB6005 carries the upstream- *rbp1*-RNP1(Rbp2)-Km^r^-downstream structure; pHB6006 carries the upstream-*rbp1*-RNP2(Rbp3)-Km^r^-downstream structure; pHB6007 carries the upstream-*rbp1*-RNP1(Rbp3)-Km^r^-downstream structure; pHB6008 carries the upstream-*rbp1*-RNP2∼RNP1(Rbp2)-Km^r^-downstream structure; pHB6009 carries the upstream-*rbp1*-RNP1∼C(Rbp2)-Km^r^-downstream structure; pHB6010 carries the upstream-*rbp1*-Km^r^-downstream structure for generating the WT (*rbp1*-K) strain.

### Purification of recombinant Rbp1 and Rbp2

Rbp1-His_6_ and Rbp2-His_6_ were expressed in *Escherichia coli* BL21 (DE3) containing pHB7083 or pHB7084 (see Table S1) by induction with 1 mM isopropyl-β-D-thiogalactopyranoside. The recombinant proteins were purified from total soluble proteins using the His·Bind purification kit (Milipore, Billerica, USA) and desalted using an Amicon Ultra filter (3-kDa cutoff; Milipore), according to the manufacturers′ instructions.

### Preparation of total RNA from *Synechocystis* 6803

Total RNA was extracted from *Synechocystis* 6803 exposed to 15°C for 2 hours using Trizol reagent (Invitrogen, Carlsbad, USA). Residual DNA was removed with DNase RQ1, and the total RNA was extracted with Trizol reagent again. After precipitation with isopropanol, RNA was washed with 70% ethanol and dissolved in nuclease-free ddH_2_O.

### RIP-seq analyses

RIP-seq (RNA-immunoprecipitation high-throughput sequencing) was performed according to Mermaz *et al*. (2018) with modifications. Magnetic beads with Rbp1 or Rbp2 were used to capture RNA from *Synechocytis* 6803. Fifty microliters of magnetic beads (MagneHis^TM^ Ni-Particles) (Promega, Madison, USA) were washed 3 times, each with 1 ml of wash buffer [20 mM Tris·Cl, pH 7.8; 150 mM NaCl; 2.5 mM MgCl_2_; 0.2% Triton X-100; 10% glycerol; 0.5 mM DTT; 1 × Protease Inhibitor Cocktail (Promega); 20 U ml^–1^ RiboLock RNase Inhibitor (Thermo Scientific, Waltham, USA)], resuspended in 50 μl of wash buffer and mixed with 0.5 mg of purified recombinant Rbp1 or Rbp2. After incubation at 4°C for 1 h, the beads were washed 3 times with the wash buffer, suspended in 1 mL of dilution buffer (20 mM Tris·Cl, pH 7.8; 150 mM NaCl; 2.5 mM MgCl_2_; 10% glycerol; 1 × Protease Inhibitor Cocktail; 20 U ml^–1^ RiboLock RNase Inhibitor), mixed with 20 μg of total RNA of *Synechocystis* 6803 and incubated at 4°C for 1 h again. The beads were then washed with 1 ml of wash buffer twice and 1 ml of dilution buffer once and treated with 50 μl of 0.04 mg ml^–1^ proteinase K in 30 mM Tris·Cl (pH 7.8) + 200 U ml^–1^ RiboLock RNase Inhibitor at 55°C for 30 min. The supernatant was transferred to a new Eppendorf tube and mixed with 150 μl of homogenization buffer (100 mM Tris·Cl, pH 7.8; 5 mM EDTA, pH 7.8; 100 mM NaCl; 0.5% SDS; 1% β-mercaptoethanol). RNA was then purified from the supernatant with Trizol reagent and precipitated with isopropanol.

Strand-specific RNA-seq libraries were prepared using the NEBNext Ultra Directional RNA Library Prep Kit for Illumina (NEB, Ipswich, USA). RNA sequencing was carried out using the Illumina NovaSeq 6000 sequencing instrument (Illumina, San Diego, USA) to generate paired-end reads with a length of 150 bp. Reads were aligned against the genome sequence of *Synechocystis* 6803 using TopHat (Trapnell *et al*. 2009). RPKM (reads per kilobase of exon per million mapped reads) values were generated from the alignment files using bam2rpkm (http://bam2rpkm.sourceforge.net). Enriched genes were identified by comparing RPKM values of the initial RNA and Rbp-bound RNA. Log_2_ Fold change ≥ 1 or ≤ -1 (*P* < 0.01) was defined as significantly increased or decreased. Data are means ± SD produced from three biological replicates. Because the RNA-binding proteins were purified from *E*. *coli* BL21, reads were also aligned against the genome sequence of *E*. *coli* BL21 to evaluate the percentage of *E*. *coli* RNA in total bound RNA.

### Western blot analyses

*Synechocystis* cells grown at 30°C were exposed to 15°C for 8 h or 48 h, collected by centrifugation, washed with 20 mM Tris·Cl (pH 8.0) and re-suspended in 20 mM Tris·Cl (pH 8.0) with 1 mM phenylmethylsulfonyl fluoride and broken by sonication. Protein concentrations were determined by the Bradford method (Kruger 2002). Proteins were separated on a 16.5% polyacrylamide gel prepared with the Tricine-SDS-PAGE (Schägger 2006) gel kit (ComWin Biotech, Beijing, China). Western blot detections were performed as previously described (Tan et al, 2011) but using rabbit antiserum against Rbp2. One hundred micrograms of proteins were loaded onto each lane; protein bands were quantified using the ImageJ software v1.53.

Quantification of Rbp1 and Rbp2 in *Synechocystis* 6803 was performed based on the linear relationship between the amount of recombinant Rbp1 or Rbp2 and the intensity of band on the Western blot. *Synechocystis* cells treated at 15°C for 2 days were collected by centrifugation at 15°C and used in the Western blot analysis. Purified recombinant proteins and *Synechocystis* proteins were detected and quantified on the same Western blot. The number of cells was counted using hemocytometer.

### Phylogenetic analysis of RNA-binding proteins

Amino acid sequences of Rbp1, Rbp2, and Rbp3 of *Synechocystis* 6803 were used as queries in BLASTP searches against all protein sequences of other eight cyanobacterial strains (Table S2). Sequence alignments were generated using MUSCLE (Edgar 2004) with default parameters, and the phylogenetic tree was constructed by MEGA 6.0 (Tamura *et al*. 2013) using the Neighbor-Joining method with 1000 bootstrap replicates.

## Results

### *In* *vitro* RNA-binding preference of Rbp1 and Rbp2

**Figure 1.**
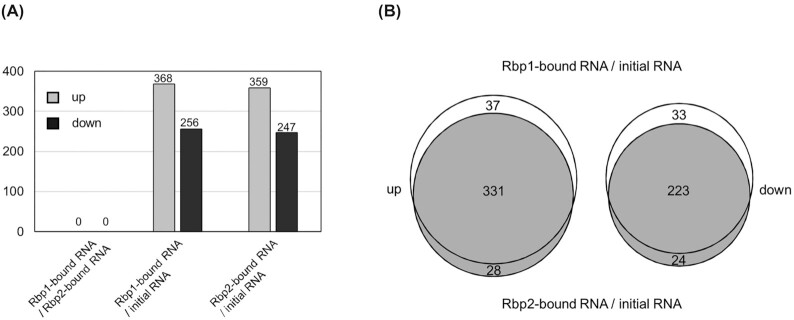
RIP-seq analysis of the RNA-binding preference of Rbp1 and Rbp2. **(A)** Numbers of genes whose transcripts showed significant differences between their abundance in Rbp1-bound RNA and that in Rbp2-bound RNA, or between Rbp1-/Rbp2-bound RNA and the initial total RNA. **(B)** Differences between Rbp1-bound RNA/initial total RNA and Rbp2-bound RNA/initial total RNA. Up (increased), Log_2_ Fold change ≥ 1; down (decreased), Log_2_ Fold change ≤ -1.

Rbp1 is required for the growth of *Synechocystis* 6803 at 15°C, whereas Rbp2 is not (Tan *et al*. 2011). Because the C-terminal glycine-rich region is lacked in Rbp2, initially we thought that this region of Rbp1 may significantly change the RNA-binding preference. To compare the RNA-binding preference of the two proteins, we performed RIP-seq analyses, in which magnetic beads with His_6_-tagged Rbp1 or Rbp2 on the surface were used to capture RNA molecules from the total RNA extracted from *Synechocytis* 6803 exposed to 15°C for 2 hours. The initial RNA pool and the captured RNA were analyzed by RNA-seq and compared with each other, and the results indicated the differences in RNA-binding preference of Rbp1 and Rbp2 under in vitro conditions. We noticed that mRNA molecules of some functionally related genes, for example, those involved in iron uptake (sll1406, sll1409, slr0513, slr1295, slr1392, slr1316-slr1317-slr1318-slr1319, slr1490, ssr2333), were significantly enriched by Rbp1 and Rbp2 on magnetic beads (Table S3). This may relate to the physiological functions of the two proteins and should be further analyzed in future. However, when Log _2_ (captured RNA/initial RNA) values were compared between Rbp1 and Rbp2, there were only some marginal differences; when the Rbp1-bound RNA and Rbp2-bound RNA were directly compared, no significant differences were found (Fig. 1; Table S3). This result implies that the C-terminal region of Rbp1 does not change the RNA preference significantly compared to Rbp2. In addition, only about 0.01% RNA-seq reads matched genes of *E*. *coli* (Rbp1 and Rbp2 were purified from *E*. *coli* cells), therefore *E*. *coli* RNA molecules should have not interfered with the binding of *Synechocystis* RNA to Rbp1 or Rbp2 on magnetic beads.

In consideration of the basically identical RNA-binding preference of Rbp1 and Rbp2 under in vitro conditions, we assumed that their roles in cold adaptability could have diverged due to the difference in cellular concentration. Using purified recombinant Rbp1 and Rbp2, we established the linear relationship between the quantity of protein and the intensity of band on the Western blot. On the same blot, Rbp1 and Rbp2 in *Synechocytis* 6803 exposed to 15°C for 48 h were quantified. We found only slight or no difference between them in cellular concentration (Fig. S1). Therefore, the role of *rbp1* in cold adaptability can't be explained by higher abundance of Rbp1 in cells either.

### Deletion of the C-terminal glycine-rich region of Rbp1 in *Synechocystis* 6803

**Figure 2.**
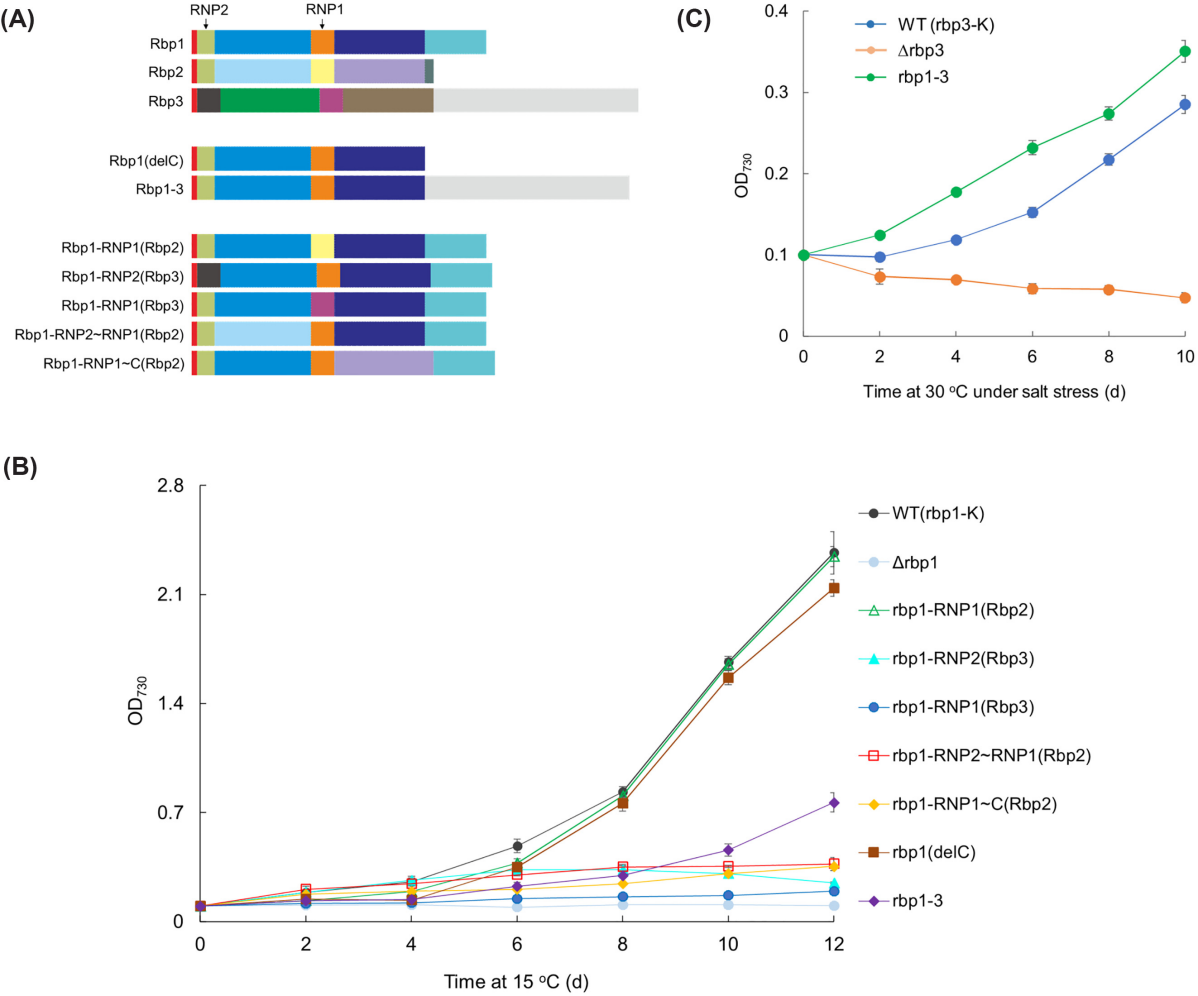
Growth of *Synechocystis* 6803 mutants at low temperature or under salt stress. **(A)** Schematic structures of Rbp1, Rbp2, Rbp3 and mutated versions of Rbp1. The 6 parts are indicated in different colors; identical sequences are indicated with the same color. **(B)** Growth of *Synechocystis* 6803 WT (*rbp1*-K) and *rbp1* mutants in BG11 at 15°C. *rbp1*-*3* here refers to the *rbp1*-*3*a strain (the C-terminal region sequence of *rbp1* was replaced with that of *rbp3* in the genome). **(C)** Growth of *Synechocystis* 6803 WT (*rbp3*-K) and *rbp3* mutants in BG11 with 1 M NaCl at 30°C. *rbp1*-*3* here refers to the *rbp1*-*3*b strain (the RRM region sequence of *rbp3* was replaced with that of *rbp1* in the genome).

**Figure 3.**
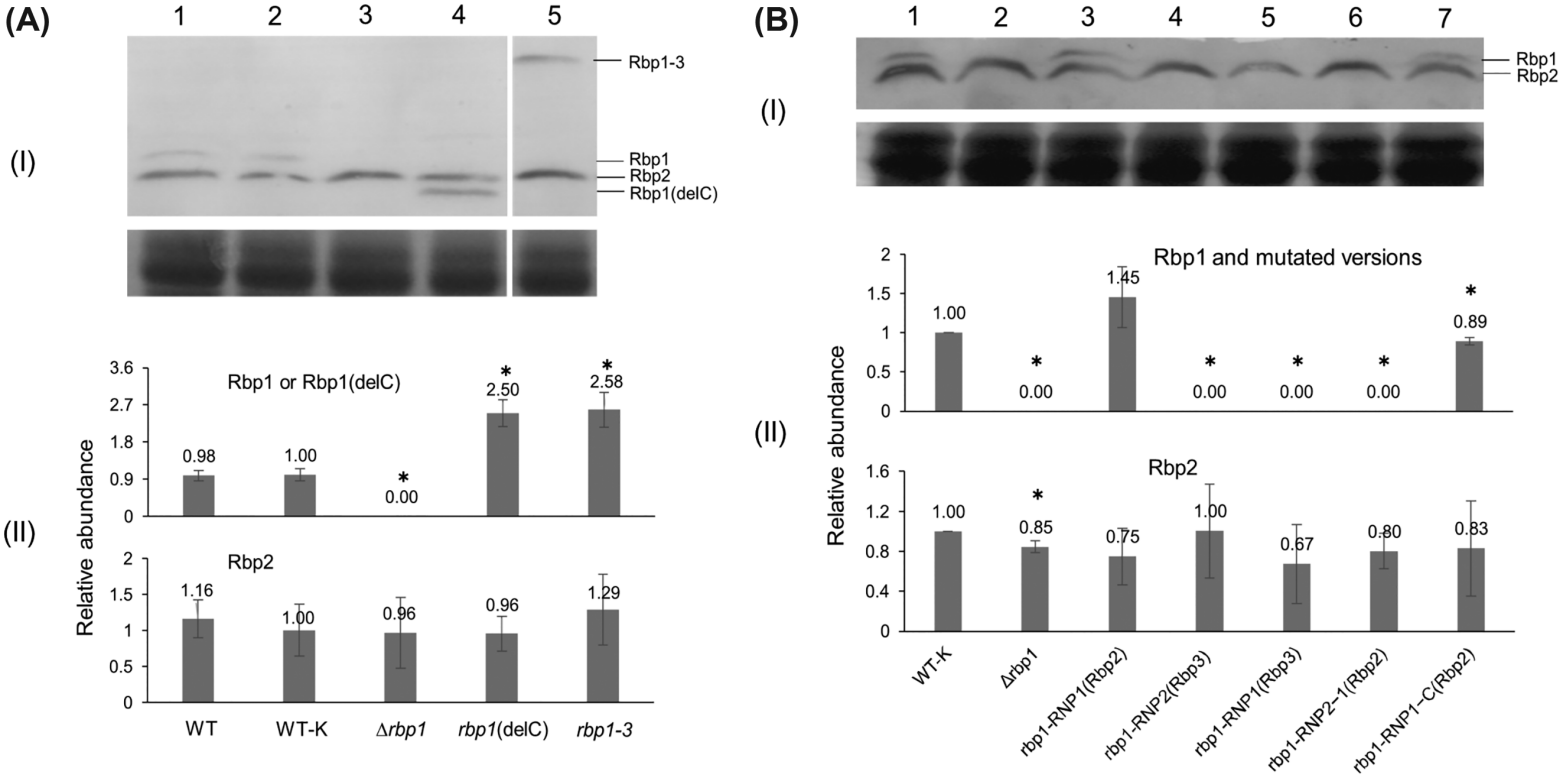
Western blot analyses of Rbp1 and mutated versions in *Synechocystis* 6803 and mutants. Cells grown at 30°C were exposed to 15°C for 8 h and used in the Western blot analyses. **(A)** and **(B)** show the effects of the C-terminal region and different parts of the RRM region on the accumulation of Rbp1 in *Synechocystis* 6803, respectively. I, Western blot detection of Rbp1 (upper) and a part of the PAGE image (lower); II, quantification of Rbp1 and Rbp2 on Western blots (three biological repeats). WT-K stands for WT (*rbp1*-K).

To test the function of the C-terminal region of Rbp1 in *Synechocystis* 6803, we generated an *rbp1* mutant in which this region was deleted in frame, as shown with Rbp1(delC) in Fig. 2A. Immediately downstream of the mutated *rbp1*, called *rbp1*(delC), was a kanamycin-resistance marker. We also generated a strain called WT (*rbp1-*K), as the ‘wild type’ control, with the kanamycin-resistance marker inserted downstream of the *rbp1* gene, and a Δ*rbp1* mutant, with the *rbp1* encoding region replaced with the kanamycin-resistance marker. Western blot detection showed the expression of Rbp1 in the WT (*rbp1-*K) strain, the truncated Rbp1 in the *rbp1*(delC) mutant, but no Rbp1 band in the Δ*rbp1* mutant (Fig. 3A). Rbp2 was expressed in all these strains at similar levels, while the abundance of Rbp1(delC) was over 2-fold that of Rbp1. Under autotrophic conditions at 30°C, all these strains showed similar growth (Fig. S2); at 15°C, the Δ*rbp1* mutant showed almost no growth, while the *rbp1*(delC) mutant grew as the wild type (Fig. 2B). These results indicated that the C-terminal region of Rbp1 was not required for the role of Rbp1 in cold adaptability of *Synechocystis* 6803.

### Functional interchangeability between sequences of Rbp1 and Rbp2 RRMs

We further checked if different parts of Rbp1 and Rbp2 RRMs were functionally interchangeable in vivo. Based on the structure, Rbp1 can be divided into 1) the N-terminal two amino acids, 2) RNP2, 3) the part between RNP2 and RNP1, 4) RNP1, 5) the part between RNP1 and the C-terminal region, and 6) the C-terminal region (Fig. 2A). We generated mutants of *Synechocystis* 6803, *rbp1*-RNP2∼RNP1(Rbp2), *rbp1*-RNP1(Rbp2) and *rbp1*-RNP1∼C(Rbp2), in which the part 3, 4, or 5 was replaced with the counterpart from Rbp2 (Fig. 2A). Parts 1 and 2 are identical between Rbp1 and Rbp2, whereas the part 6 is lacked in Rbp2. We also generated mutants *rbp1*-RNP2(Rbp3) and *rbp1*-RNP1(Rbp3), in which RNP2 or RNP1 was replaced with that from Rbp3 (Fig. 2A), as additional controls. Western blot detection showed the expression of Rbp1-RNP1(Rbp2) and Rbp1-RNP1∼C(Rbp2) but no band for other forms of mutated Rbp1 (Fig. 3B). The abundance of Rbp1-RNP1(Rbp2) and Rbp1-RNP1∼C(Rbp2) was close to that of Rbp1. Tests of growth at 15°C indicated that only Rbp1-RNP1(Rbp2) fully retained the function of Rbp1, while all the others were not functional (Fig. 2B). These results indicated that the sequence between RNP2 and RNP1 and that between RNP1 and the C-terminal region may determine the functional divergence between Rbp1 and Rbp2 in *Synechocystis*.

### Directing the function of Rbp1 RRM with the C-terminal region of Rbp3

Unlike Rbp1 and Rbp2, Rbp3 possesses a longer C-terminal region which is not rich in glycine. We wondered whether the function of Rbp1 can be changed by the C-terminal sequence from Rbp3. Thus, the *rbp1-3*a mutant of *Synechocystis* 6803 was generated, in which the C-terminal glycine-rich region of Rbp1 was replaced with the long C-terminal region of Rbp3 (Rbp1-3 in Fig. 2A). The abundance of Rbp1-3a was more than 2-fold that of Rbp1 (Fig. 2A). This mutant showed much slower growth at 15°C than WT-*rbp1*K and the *rbp1*(delC) mutant (Fig. 2B). Apparently, Rbp1-3 only retained the partial function of Rbp1. Because the C-terminal half of Rbp1-3 was from Rbp3, we tested the function of such a fusion protein in salt tolerance as well. The *rbp1-3*b mutant of *Synechocystis* 6803 was therefore generated, in which the RRM portion of Rbp3 was replaced with that from Rbp1. WT (*rbp3*-K) and the Δ*rbp3* mutant were generated as the ‘wild type’ and the negative control. In BG11 with 1 M NaCl, the *rbp1-3*b mutant showed even better growth than the WT (*rbp3*-K) strain, while the Δ*rbp3* mutant was dying, with OD_730nm_ gradually decreased (Fig. 2C); without the salt stress, these strains grew similarly (Fig. S3). Therefore, Rbp1-3 fully retained the function of Rbp3 in salt tolerance. The improved growth of the *rbp1-3*b mutant under the salt stress could be due to the higher abundance of Rbp1-3 in this mutant than that of Rbp3 in WT (*rbp3*-K) (data not shown).

### Classification of RRM-type RNA-binding proteins in cyanobacteria

**Figure 4.**
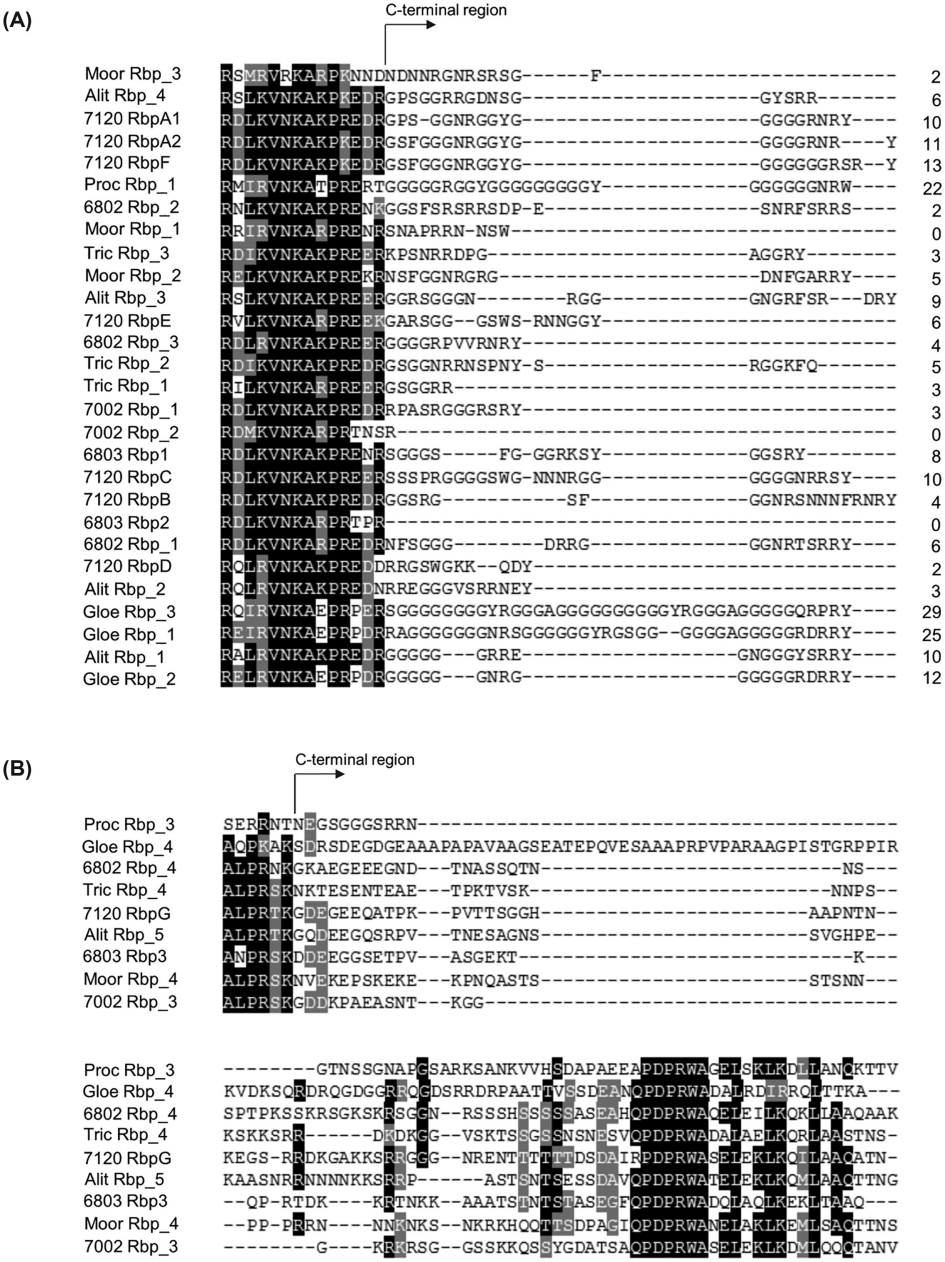
Alignments of partial RRM and C-terminal region sequences of the cluster I **(A)** and cluster II **(B)** RNA-binding proteins in the phylogenetic tree. Except for the C-terminal regions of proteins in (A), sequence similarities are indicated in dark (identical) and grey (positive). Numbers of glycine residues in the C-terminal region are indicated next to each sequence in (A). Alit, *Aliterella atlantica* CENA595; Gloe, *Gloeobacter violaceus* PCC 7421; Moor, *Moorea* sp. SIO3E8; Proc, *Prochlorococcus marinus* MIT9319, Tric, *Trichodesmium erythraeum* IMS101; 6802, *Pseudanabaena* sp. PCC 6802; 6803, *Synechocystis* sp. PCC 6803; 7002, *Synechococcus* sp. PCC 7002; 7120, *Anabaena* sp. PCC 7120.

Because the C-terminal glycine-rich region is not required for the function of Rbp1, we re-visited the question how RRM RNA-binding proteins in cyanobacteria should be classified. Nine species/strains that represent typical cyanobacterial groups were chosen for the analysis, including *Aliterella atlantica* CENA595, *Anabaena* sp. PCC 7120, *Gloeobacter violaceus* PCC 7421, *Moorea* sp. SIO3E8, *Prochlorococcus marinus* MIT9313, *Pseudanabaena* sp. PCC 6802, *Synechocystis* sp. PCC 6803, *Synechococcus* sp. PCC 7002, *Trichodesmium erythraeum* IMS101. As previously reported, there are two major clusters in the phylogenetic tree of RRM RNA-binding proteins (Fig. S4): cluster I, Rbps with or without a C-terminal glycine-rich region (see Fig. 4A), such as Rbp1 and Rbp2; cluster II, Rbps with a conserved C-terminal region (in particular the PDPRWA motif), such as Rbp3 (Fig. 4B). Apparently, cluster I Rbps should not be divided into a group with, and a group without, the C-terminal glycine-rich region. As shown in Fig. 4A, among the 28 cluster I Rbps, there are 3 with no glycine residue in the C-terminal region (or without this region), 14 with 2 to 6 glycine residues, 8 with 8 to 12, 3 with more than 20. Rbp_2 of *Prochlorococcus* sp. MIT9313, which is not included in Fig. 4A, possesses 66 glycine residues in the 116-aa long C-terminal region (too long to be included in the figure).

## Discussion

In *Synechocystis* 6803, Rbp1 plays an important role in cold adaptability, whereas Rbp2 does not. The most prominent structural difference between the two proteins appears to be the presence of the C-terminal glycine-rich region or not. In this study, we showed that this region is not required for the function of Rbp1. First, in vitro RNA-binding preference of recombinant Rbp1 and Rbp2 were virtually identical (Fig. 1 and Table S3); second, the in-frame deletion of the C-terminal glycine-rich region from the Rbp1-encoding gene did not alter the growth capability of *Synechocystis* 6803 at the low temperature (Fig. 2B). So, which portions of the RRM RNA-binding protein determine the specific role of Rbp1, compared to Rbp2, in cold adaptability? Further experiments indicated that the portion between RNP2 and RNP1 and that between RNP1 and the C-terminal region are critical for the accumulation and function of Rbp1 in *Synechocystis* 6803 (Fig. 2B). Hypothetically, Rbp1 and Rbp2 may differ from each other in modifications, interactions with other molecules (such as proteins, degradosome, metabolites, etc.) or internal structural compatibility, and the differences are based on the divergences between these portions of Rbp1 and the counterparts of Rbp2.

Unlike the C-terminal region of Rbp1, that of Rbp3 appears to play a critical role in directing the function of the RNA-binding protein: a combination of the Rbp1 RRM region with the long ‘tail’ of Rbp3 resulted in a protein that functioned as Rbp3 in salt tolerance (Fig. 2C). In contrast, the same protein was basically unable to function as Rbp1 in cold adaptability (Fig. 2B). The C-terminal region from Rbp3 apparently directed the Rbp1 RRM to a different function, presumably through alteration of the post-transcriptional regulation profile of the RNA-binding protein.

In the phylogenetic tree, cyanobacterial RRM-type RNA-binding proteins form two clusters. In the past literatures, the cluster I RNA binding proteins were further divided into two types based on the C-terminal glycine-rich region and the conserved boxes for cold response in the 5′-UTR of the gene (Maruyama *et al*. 1999, Hamano *et al*. 2004). The presence of conserved boxes in the 5′-UTR is apparently not a reliable criterion. In this study, we further showed that the C-terminal glycine-rich region should not be taken as a criterion for classification either, because it is not required for the role of Rbp1 in cold adaptability, and the numbers of glycine residues in most cluster I Rbps constitute an essentially continual spectrum (from 0 to 12) (Fig. 4A). We don't exclude the possibility that the C-terminal regions of other cluster I members are involved in certain physiological functions, but it is clear that the C-terminal glycine-rich region is not an adequate criterion for classification.

Based on all the bioinformatic and experimental analyses, we propose that the RRM-type RNA binding proteins in cyanobacteria should be only classified as two types. Type I: Rbps of this type typically possess a short C-terminal glycine-rich region (usually less than 45 aa) or not, represented by Rbp1 and Rbp2 in *Synechocystis* 6803, but some in *Prochlorococcus* possess a C-terminal glycine-rich region up to around 130 aa; type II: Rbps of this type possess a relatively long C-terminal region (usually more than 60 aa) with the PDPRWA motif, represented by Rbp3 in *Synechocystis* 6803. These two types correspond to the previously proposed two classes (Hamano *et al*. 2004), but type I in our classification is not further divided on the basis of C-terminal sequences.

## Supplementary Material

fnac021_Supplemental_Files

## References

[bib1] Castenholz RW . Oxygenic photosynthetic bacteria. In: Boone DR, Castenholz RW (eds). Bergey's Manual of Systematic Bacteriology. New York, Berlin, Heidelberg: Springer-Verlag, 2001, 474–87.

[bib2] Cléry A , Blatter M, Allain FH. RNA recognition motifs: boring? Not quite. Curr Opin Struct Biol. 2008;18:290–8. 18515081 10.1016/j.sbi.2008.04.002

[bib3] Corley M , Burns MC, Yeo GW. How RNA-binding proteins interact with RNA: molecules and mechanisms. Mol Cell. 2020;78:9–29. 32243832 10.1016/j.molcel.2020.03.011 PMC7202378

[bib4] Daubner GM , Cléry A, Allain FH. RRM-RNA recognition: NMR or crystallography and new findings. Curr Opin Struct Biol. 2013;23:100–8. 23253355 10.1016/j.sbi.2012.11.006

[bib5] Edgar RC . MUSCLE: multiple sequence alignment with high accuracy and high throughput. Nucleic Acids Res. 2004;32:1792–7. 15034147 10.1093/nar/gkh340 PMC390337

[bib6] Flombaum P , Gallegos JL, Gordillo RA et al. Present and future global distributions of the marine Cyanobacteria *Prochlorococcus* and *Synechococcu**s*. Proc Natl Acad Sci. 2013;110:9824–9. 23703908 10.1073/pnas.1307701110 PMC3683724

[bib7] Glisovic T , Bachorik JL, Yong J et al. RNA-binding proteins and post-transcriptional gene regulation. FEBS Lett. 2008;582:1977–86. 18342629 10.1016/j.febslet.2008.03.004 PMC2858862

[bib8] Hamano T , Murakami S, Takayama K et al. Characterization of RNA-binding properties of three types of RNA-binding proteins in *Anabaena* sp. PCC 7120. Cell Mol Biol (Noisy-le-grand). 2004;50:613–24. 15559978

[bib9] Holmqvist E , Vogel J. RNA-binding proteins in bacteria. Nat Rev Microbiol. 2018;16:601–15. 29995832 10.1038/s41579-018-0049-5

[bib10] Hutchins DA , Fu F. Microorganisms and ocean global change. Nature Microbiology. 2017;2:17058. 10.1038/nmicrobiol.2017.58 28540925

[bib11] Kruger NJ . The Bradford method for protein quantitation. In: Walker JM (ed). The protein protocols handbook(2nd edition). Totowa: Humana Press, 2002, 15–21.

[bib12] Mahbub M , Hemm L, Yang Y et al. mRNA localization, reaction center biogenesis and thylakoid membrane targeting in cyanobacteria. Nature Plants. 2020;6:1179–91. 32895528 10.1038/s41477-020-00764-2

[bib13] Maruyama K , Sato N, Ohta N. Conservation of structure and cold-regulation of RNA-binding proteins in cyanobacteria: probable convergent evolution with eukaryotic glycine-rich RNA-binding proteins. Nucleic Acids Res. 1999;27:2029–36. 10198437 10.1093/nar/27.9.2029 PMC148417

[bib14] Mermaz B , Liu F, Song J. RNA immunoprecipitation protocol to identify protein-RNA interactions in *Arabidopsis**thaliana*. In: Marian B, Célia B (eds). Plant Chromatin Dynamics. New York: Humana Press, 2018, 331–43. 10.1007/978-1-4939-7318-7_19 29052200

[bib15] Ng Kwan Lim E , Sasseville C, Carrier MC et al. Keeping up with RNA-Based regulation in bacteria: new roles for RNA binding proteins. Trends Genet. 2021;37:86–97. 33077249 10.1016/j.tig.2020.09.014

[bib16] Sato N , Wada A. Disruption analysis of the gene for a cold-regulated RNA-binding protein, *rbpA1*, in *Anabaena*: cold-induced initiation of the heterocyst differentiation pathway. Plant Cell Physiol. 1996;37:1150–60. 9032967 10.1093/oxfordjournals.pcp.a029066

[bib17] Schägger H . Tricine–SDS-PAGE. Nat Protoc. 2006;1:16–22. 17406207 10.1038/nprot.2006.4

[bib18] Tamura K , Stecher G, Peterson D et al. MEGA6: molecular evolutionary genetics analysis version 6.0. Mol Biol Evol. 2013;30:2725–9. 24132122 10.1093/molbev/mst197 PMC3840312

[bib19] Tan X , Zhu T, Shen S et al. Role of Rbp1 in the acquired chill-light tolerance of cyanobacteria. J Bacteriol. 2011;193:2675–83. 21460086 10.1128/JB.01454-10 PMC3133111

[bib20] Trapnell C , Pachter L, Salzberg SL. TopHat: discovering splice junctions with RNA-Seq. Bioinformatics. 2009;25:1105–11. 19289445 10.1093/bioinformatics/btp120 PMC2672628

[bib21] Wang Y , Xu X. Effects of Rbp3 on lipid peroxidation and salt tolerance in *Synechocysti*s sp. PCC 6803. FEBS Lett. 2013;587:1446–51. 23542033 10.1016/j.febslet.2013.03.028

[bib22] Williams JGK . Construction of specific mutations in photosystem II photosynthetic reaction center by engineering methods in *Synechocystis* 6803. Methods Enzymol. 1988;167:766–78.

