# Supplementary material for: Two types of C-terminal regions of RNA-binding proteins play distinct roles in stress tolerance of Synechocystis sp. PCC 6803
Source: FEMS Microbiol Lett. 2022 Feb 25;369(1):fnac021. doi: 10.1093/femsle/fnac021 (PMC9333190; doi:10.1093/femsle/fnac021)
Supplement: fnac021_Supplemental_Files [file fnac021_supplemental_files.zip › Table_S3.pdf]

Table S3 RIP-seq analysis of the RNA-binding preference of Rbp1 and Rbp2

| Gene ID | Rbp1-bound RNA/Initial RNA | | Rbp2-bound RNA/Initial RNA | | Annotation |
|---|---|---|---|---|---|
| | $log_2$ Fold Change | Padj | $log_2$ Fold Change | Padj | |
| sll0248 | 9.515 | 0.000 | 9.457 | 0.000 | flavodoxin |
| sll0249 | 8.542 | 0.000 | 8.543 | 0.000 | hypothetical protein |
| sll1549 | 7.115 | 0.000 | 7.170 | 0.000 | hypothetical protein |
| sll1405 | 6.167 | 0.000 | 6.149 | 0.000 | biopolymer transporter ExbD |
| sll1404 | 5.967 | 0.000 | 5.930 | 0.000 | biopolymer transporter ExbB |
| sll1408 | 5.387 | 0.000 | 5.389 | 0.000 | AraC family transcriptional regulator |
| sll1407 | 5.375 | 0.000 | 5.383 | 0.000 | SAM-dependent methyltransferase |
| slr1485 | 5.071 | 0.000 | 4.845 | 0.000 | hypothetical protein |
| slr1295 | 4.930 | 0.000 | 4.891 | 0.000 | iron uptake protein A1 |
| sll1406 | 4.884 | 0.000 | 4.774 | 0.000 | TonB-dependent siderophore receptor |
| slr1488 | 4.877 | 0.000 | 5.003 | 0.000 | helicase |
| ssr2333 | 4.612 | 0.000 | 4.381 | 0.000 | iron ABC transporter |
| slr0513 | 4.483 | 0.000 | 4.497 | 0.000 | iron uptake protein A2 |
| ssl0461 | 4.304 | 0.000 | 4.274 | 0.000 | hypothetical protein |
| sll0247 | 4.295 | 0.000 | 4.307 | 0.000 | iron stress-induced chlorophyll-binding protein |
| slr1392 | 4.261 | 0.000 | 4.210 | 0.000 | ferrous iron transporter B |
| slr1484 | 3.933 | 0.000 | 3.938 | 0.000 | hypothetical protein |
| sll0250 | 3.846 | 0.000 | 3.723 | 0.000 | pantothenate metabolism flavoprotein |
| slr1068 | 3.725 | 0.000 | 3.751 | 0.000 | hypothetical protein |
| sll1202 | 3.677 | 0.000 | 3.700 | 0.000 | hypothetical protein |

| | | | | | |
|---|---|---|---|---|---|
| slr1069 | 3.470 | 0.000 | 3.619 | 0.000 | hypothetical protein |
| slr1318 | 3.463 | 0.000 | 3.716 | 0.000 | iron-enterobactin transporter ATP-binding protein |
| slr1316 | 3.388 | 0.000 | 3.260 | 0.000 | iron(III) dicitrate transport system permease protein; FecC |
| slr6016 | 3.387 | 0.000 | 3.224 | 0.000 | unknown protein |
| slr6075 | 3.387 | 0.000 | 3.224 | 0.000 | unknown protein |
| sll0720 | 3.383 | 0.000 | 3.383 | 0.000 | toxin-activating lysine-acyltransferase |
| sll7062 | 3.319 | 0.000 | 3.196 | 0.000 | hypothetical protein |
| slr6014 | 3.236 | 0.000 | 3.159 | 0.000 | unknown protein |
| slr6073 | 3.236 | 0.000 | 3.159 | 0.000 | unknown protein |
| sll1198 | 3.230 | 0.000 | 2.968 | 0.000 | tRNA (guanine-N1)-methyltransferase |
| sll1203 | 3.132 | 0.000 | 3.344 | 0.000 | hypothetical protein |
| slr1317 | 3.121 | 0.000 | 3.175 | 0.000 | iron ABC transporter permease |
| sll1550 | 3.109 | 0.000 | 3.133 | 0.000 | membrane protein |
| ssr7084 | 3.097 | 0.000 | 3.369 | 0.000 | unknown protein |
| slr1070 | 3.067 | 0.000 | 2.960 | 0.000 | unknown protein |
| sll1409 | 2.971 | 0.000 | 2.929 | 0.000 | TonB-dependent siderophore receptor |
| slr1074 | 2.969 | 0.000 | 2.852 | 0.000 | unknown protein |
| sll1205 | 2.934 | 0.000 | 3.034 | 0.000 | AraC family transcriptional regulator |
| slr6013 | 2.890 | 0.000 | 2.906 | 0.000 | unknown protein |
| slr6072 | 2.890 | 0.000 | 2.906 | 0.000 | unknown protein |
| sll7085 | 2.886 | 0.000 | 2.848 | 0.000 | hypothetical protein |
| sll1206 | 2.849 | 0.000 | 2.933 | 0.000 | ligand-gated channel |
| slr6015 | 2.826 | 0.000 | 2.742 | 0.000 | unknown protein |
| slr6074 | 2.826 | 0.000 | 2.742 | 0.000 | unknown protein |
| slr0514 | 2.820 | 0.000 | 2.766 | 0.000 | hypothetical protein |

| | | | | | |
|---|---|---|---|---|---|
| sll1552 | 2.795 | 0.000 | 2.747 | 0.000 | GNAT family N-acetyltransferase |
| ssl2250 | 2.773 | 0.000 | 2.854 | 0.000 | glycoprotein |
| slr1071 | 2.773 | 0.000 | 2.646 | 0.000 | unknown protein |
| slr6104 | 2.771 | 0.000 | 2.752 | 0.000 | hypothetical protein |
| slr1066 | 2.758 | 0.000 | 2.782 | 0.000 | unknown protein |
| slr6100 | 2.701 | 0.000 | 2.281 | 0.000 | hypothetical protein |
| sll7063 | 2.592 | 0.000 | 2.615 | 0.000 | CRISPR-associated RAMP family protein |
| slr1489 | 2.574 | 0.000 | 2.462 | 0.000 | AraC family transcriptional regulator |
| sml0009 | 2.548 | 0.000 | 2.219 | 0.000 | similar to virulence-associated protein VapC |
| slr5085 | 2.540 | 0.008 | 1.935 | 0.098 | unknown protein |
| slr1247 | 2.508 | 0.000 | 2.591 | 0.000 | phosphate ABC transporter substrate-binding protein PstS |
| slr1248 | 2.493 | 0.000 | 2.557 | 0.000 | phosphate ABC transporter permease subunit PstC |
| sll0656 | 2.454 | 0.000 | 2.454 | 0.000 | nuclease |
| sll7086 | 2.452 | 0.000 | 2.295 | 0.000 | hypothetical protein |
| slr2110 | 2.445 | 0.000 | 2.269 | 0.000 | unknown protein |
| slr1063 | 2.369 | 0.000 | 2.208 | 0.000 | probable glycosyltransferase |
| sll0710 | 2.367 | 0.000 | 2.324 | 0.000 | unknown protein |
| slr7088 | 2.349 | 0.000 | 2.211 | 0.000 | peptidase |
| slr5016 | 2.343 | 0.000 | 2.339 | 0.000 | unknown protein |
| sll0447 | 2.328 | 0.000 | 2.317 | 0.000 | unknown protein |
| slr2006 | 2.326 | 0.000 | 2.482 | 0.000 | cation:proton antiporter |
| sll0477 | 2.306 | 0.000 | 2.262 | 0.000 | biopolymer transporter ExbB |
| slr6051 | 2.261 | 0.000 | 2.125 | 0.000 | hypothetical protein |
| slr1319 | 2.258 | 0.000 | 2.132 | 0.000 | iron siderophore-binding protein |
| slr1065 | 2.258 | 0.000 | 2.202 | 0.000 | probable glycosyltransferase |

| | | | | | |
|---|---|---|---|---|---|
| slr6050 | 2.258 | 0.000 | 2.253 | 0.000 | hypothetical protein |
| sll0722 | 2.237 | 0.000 | 2.244 | 0.000 | unknown protein |
| ssr2227 | 2.235 | 0.000 | 1.794 | 0.000 | putative transposase |
| slr1067 | 2.200 | 0.000 | 2.159 | 0.000 | UDP-glucose 4-epimerase |
| sll0709 | 2.188 | 0.000 | 2.125 | 0.000 | putative endonuclease |
| slr1243 | 2.186 | 0.000 | 2.103 | 0.000 | unknown protein |
| slr1135 | 2.177 | 0.000 | 1.870 | 0.000 | hypothetical protein |
| sll0721 | 2.166 | 0.000 | 2.101 | 0.000 | unknown protein |
| sll7066 | 2.155 | 0.000 | 2.124 | 0.000 | unknown protein |
| sll0922 | 2.147 | 0.000 | 2.236 | 0.000 | unknown protein |
| sll7064 | 2.141 | 0.000 | 2.170 | 0.000 | unknown protein |
| slr1064 | 2.137 | 0.000 | 2.163 | 0.000 | probable glycosyltransferase |
| sll0479 | 2.135 | 0.000 | 1.747 | 0.000 | biopolymer transporter ExbD |
| slr1250 | 2.120 | 0.000 | 2.064 | 0.000 | phosphate transport ATP-binding protein PstB homolog |
| sml0010 | 2.117 | 0.003 | 1.884 | 0.015 | putative transposase |
| sll7087 | 2.104 | 0.000 | 1.990 | 0.000 | type III-B CRISPR module RAMP protein Cmr4 |
| sll7065 | 2.103 | 0.000 | 2.038 | 0.000 | unknown protein |
| slr0607 | 2.099 | 0.000 | 2.106 | 0.000 | hypothetical protein |
| sll1429 | 2.079 | 0.000 | 1.847 | 0.000 | unknown protein |
| slr1863 | 2.069 | 0.000 | 2.184 | 0.000 | unknown protein |
| slr2117 | 2.067 | 0.000 | 1.795 | 0.000 | hypothetical protein |
| sll0478 | 2.030 | 0.000 | 1.885 | 0.000 | biopolymer transporter ExbD |
| slr1862 | 1.992 | 0.000 | 2.023 | 0.000 | unknown protein |
| slr6012 | 1.976 | 0.000 | 1.901 | 0.000 | unknown protein |
| slr6071 | 1.976 | 0.000 | 1.901 | 0.000 | unknown protein |

| | | | | | |
|---|---|---|---|---|---|
| slr1861 | 1.976 | 0.000 | 2.135 | 0.000 | probable sigma regulatory factor |
| slr1915 | 1.934 | 0.000 | 1.889 | 0.000 | hypothetical protein |
| slr2116 | 1.934 | 0.000 | 1.589 | 0.000 | probable glycosyltransferase |
| ssl2923 | 1.933 | 0.000 | 1.698 | 0.000 | similar to virulence-associated protein VapC |
| sll6109 | 1.920 | 0.000 | 1.956 | 0.000 | hypothetical protein |
| slr7005 | 1.917 | 0.000 | 1.991 | 0.000 | integrase/recombinase |
| slr1249 | 1.913 | 0.000 | 1.870 | 0.000 | phosphate ABC transporter, permease protein PstA |
| slr1859 | 1.885 | 0.000 | 1.754 | 0.000 | anti-sigma f factor antagonist |
| slr1851 | 1.860 | 0.000 | 1.669 | 0.000 | hypothetical protein |
| sll1878 | 1.858 | 0.000 | 1.918 | 0.000 | ABC transporter |
| slr5112 | 1.858 | 0.000 | 1.734 | 0.000 | hypothetical protein |
| sll1503 | 1.849 | 0.000 | 1.729 | 0.000 | unknown protein |
| sll0594 | 1.846 | 0.000 | 1.688 | 0.000 | regulatory protein CysR |
| slr1073 | 1.839 | 0.000 | 1.822 | 0.000 | unknown protein |
| slr1056 | 1.820 | 0.000 | 1.725 | 0.000 | unknown protein |
| sll0448 | 1.809 | 0.000 | 1.686 | 0.000 | unknown protein |
| sll7103 | 1.808 | 0.000 | 1.375 | 0.005 | exodeoxyribonuclease V, alpha chain |
| slr0913 | 1.798 | 0.000 | 1.761 | 0.000 | unknown protein |
| sll7067 | 1.794 | 0.000 | 1.777 | 0.000 | unknown protein |
| slr2126 | 1.789 | 0.000 | 1.680 | 0.000 | probable glycosyltransferase |
| slr1860 | 1.788 | 0.000 | 1.704 | 0.000 | carbon metabolisms regulatory protein IcfG |
| slr0376 | 1.783 | 0.000 | 1.818 | 0.000 | hypothetical protein |
| slr1397 | 1.775 | 0.000 | 1.868 | 0.000 | unknown protein |
| ssl0511 | 1.771 | 0.000 | 1.690 | 0.000 | hypothetical protein |
| slr0587 | 1.769 | 0.000 | 1.504 | 0.000 | unknown protein |

| | | | | | |
|---|---|---|---|---|---|
| slr6108 | 1.752 | 0.000 | 1.637 | 0.000 | hypothetical protein |
| ssr0335 | 1.741 | 0.000 | 1.830 | 0.000 | unknown protein |
| slr6029 | 1.734 | 0.000 | 1.755 | 0.000 | hypothetical protein |
| slr6088 | 1.734 | 0.000 | 1.755 | 0.000 | hypothetical protein |
| sll1600 | 1.729 | 0.000 | 1.585 | 0.000 | manganese transport system membrane protein MntB |
| slr0732 | 1.726 | 0.000 | 1.453 | 0.000 | hypothetical protein |
| slr7025 | 1.725 | 0.000 | 1.623 | 0.000 | hypothetical protein |
| slr0871 | 1.721 | 0.001 | 1.600 | 0.002 | hypothetical protein |
| sll1504 | 1.719 | 0.000 | 1.536 | 0.000 | hypothetical protein |
| slr1651 | 1.718 | 0.000 | 1.728 | 0.000 | ABC transporter ATP-binding protein |
| slr5029 | 1.715 | 0.000 | 1.630 | 0.000 | putative transposase [ISY391d(partial copy): 31333 - 32206] |
| ssr6099 | 1.712 | 0.000 | 1.603 | 0.000 | unknown protein |
| slr1210 | 1.686 | 0.000 | 1.417 | 0.001 | unknown protein |
| sll0371 | 1.673 | 0.000 | 1.445 | 0.000 | unknown protein |
| ssr1260 | 1.667 | 0.000 | 1.475 | 0.000 | hypothetical protein |
| ssr0336 | 1.656 | 0.000 | 1.417 | 0.000 | hypothetical protein |
| slr6021 | 1.649 | 0.000 | 1.091 | 0.001 | unknown protein |
| slr6080 | 1.649 | 0.000 | 1.091 | 0.001 | unknown protein |
| slr2118 | 1.646 | 0.000 | 1.755 | 0.000 | unknown protein |
| slr1866 | 1.645 | 0.000 | 1.688 | 0.000 | unknown protein |
| sll5041 | 1.635 | 0.000 | 1.483 | 0.000 | transposase (plasmid) |
| sll1204 | 1.632 | 0.000 | 1.710 | 0.000 | similar to macrolide efflux protein |
| sll1599 | 1.628 | 0.000 | 1.611 | 0.000 | manganese transport system ATP-binding protein MntA |
| slr0656 | 1.627 | 0.000 | 1.620 | 0.000 | hypothetical protein |
| sll1505 | 1.622 | 0.000 | 1.577 | 0.000 | hypothetical protein |

| | | | | | |
|---|---|---|---|---|---|
| sll5097 | 1.618 | 0.000 | 1.640 | 0.000 | hypothetical protein |
| sll1594 | 1.615 | 0.000 | 1.583 | 0.000 | LysR family transcriptional regulator |
| ssl5098 | 1.607 | 0.000 | 1.702 | 0.000 | unknown protein |
| slr5111 | 1.607 | 0.000 | 1.640 | 0.000 | unknown protein |
| slr0983 | 1.600 | 0.000 | 1.625 | 0.000 | glucose-1-phosphate cytidylyltransferase |
| sll0688 | 1.597 | 0.000 | 1.296 | 0.000 | unknown protein |
| ssl7045 | 1.593 | 0.004 | 1.049 | 0.119 | unknown protein |
| slr0746 | 1.590 | 0.000 | 1.477 | 0.000 | glucosylglycerolphosphate phosphatase |
| sll1471 | 1.590 | 0.000 | 1.678 | 0.000 | phycobilisome rod-core linker polypeptide |
| sll7089 | 1.590 | 0.000 | 1.334 | 0.000 | hypothetical protein |
| slr8045 | 1.588 | 0.000 | 1.506 | 0.000 | putative transposase [ISY100x: 40984 - 41929] |
| ssr6048 | 1.580 | 0.000 | 1.482 | 0.000 | unknown protein |
| sll1879 | 1.576 | 0.000 | 1.584 | 0.000 | hypothetical protein |
| sll8043 | 1.563 | 0.000 | 1.493 | 0.000 | putative transposase [ISY100y: 38542 - 39487] |
| slr1285 | 1.550 | 0.000 | 1.495 | 0.000 | two-component sensor histidine kinase |
| ssl1507 | 1.545 | 0.000 | 1.404 | 0.000 | putative transposase [ISY508a: 1710788 - 1711753] |
| slr6047 | 1.542 | 0.000 | 1.532 | 0.000 | hypothetical protein |
| sll1401 | 1.541 | 0.000 | 1.624 | 0.000 | unknown protein |
| sll0910 | 1.536 | 0.000 | 1.370 | 0.000 | unknown protein |
| slr0460 | 1.531 | 0.000 | 1.349 | 0.000 | putative transposase [ISY352g: 3511668 - 3512290, join 3513238 - 3514051] |
| sll0243 | 1.531 | 0.000 | 1.441 | 0.000 | unknown protein |
| sll1851 | 1.526 | 0.001 | 1.560 | 0.001 | unknown protein |
| slr1977 | 1.524 | 0.000 | 1.365 | 0.000 | hypothetical protein |
| slr6097 | 1.523 | 0.000 | 1.420 | 0.000 | type I site-specific deoxyribonuclease |

| | | | | | |
|---|---|---|---|---|---|
| slr1072 | 1.521 | 0.000 | 1.509 | 0.000 | GDP-D-mannose dehydratase |
| slr7071 | 1.520 | 0.000 | 1.398 | 0.000 | CRISPR-associated endonuclease Cas1 |
| sll6017 | 1.518 | 0.000 | 1.386 | 0.000 | hypothetical protein |
| sll6076 | 1.518 | 0.000 | 1.386 | 0.000 | hypothetical protein |
| ssr8047 | 1.505 | 0.002 | 1.456 | 0.002 | unknown protein |
| ssr3159 | 1.502 | 0.000 | 1.391 | 0.000 | unknown protein |
| ssl0323 | 1.496 | 0.001 | 1.124 | 0.020 | unknown protein |
| ssl2920 | 1.493 | 0.000 | 1.403 | 0.000 | hypothetical protein |
| ssr7036 | 1.487 | 0.001 | 1.580 | 0.000 | unknown protein |
| sll0494 | 1.484 | 0.000 | 1.587 | 0.000 | unknown protein |
| sll0654 | 1.472 | 0.000 | 1.480 | 0.000 | alkaline phosphatase |
| slr2115 | 1.469 | 0.000 | 1.565 | 0.000 | unknown protein |
| slr6103 | 1.468 | 0.000 | 1.513 | 0.000 | hypothetical protein |
| slr0516 | 1.466 | 0.000 | 1.749 | 0.000 | hypothetical protein |
| ssl2922 | 1.460 | 0.000 | 1.234 | 0.000 | similar to virulence-associated protein VapB |
| slr1396 | 1.439 | 0.000 | 1.401 | 0.000 | unknown protein |
| sll0670 | 1.438 | 0.001 | 0.927 | 0.031 | hypothetical protein |
| slr0392 | 1.437 | 0.000 | 1.393 | 0.000 | unknown protein |
| slr0393 | 1.435 | 0.000 | 1.591 | 0.000 | unknown protein |
| slr0869 | 1.429 | 0.000 | 1.353 | 0.000 | hypothetical protein |
| slr1610 | 1.429 | 0.000 | 1.186 | 0.000 | putative C-3 methyl transferase |
| slr0985 | 1.428 | 0.000 | 1.361 | 0.000 | dTDP-4-dehydrorhamnose 3,5-epimerase |
| sll0776 | 1.427 | 0.000 | 1.416 | 0.000 | serine/threonine kinase |
| slr1617 | 1.422 | 0.000 | 1.350 | 0.000 | similar to UDP-glucose 4-epimerase |
| slr1283 | 1.421 | 0.000 | 0.886 | 0.032 | putative transposase [ISY508b: 1877114 - 1878081] |

| | | | | | |
|---|---|---|---|---|---|
| sll0317 | 1.420 | 0.000 | 1.450 | 0.000 | putative transposase [ISY203i: 2443391 - 2443924, join 2444874 - 2445513] |
| sll1982 | 1.420 | 0.000 | 1.175 | 0.000 | putative transposase [ISY352c: 1553414 - 1553903, join 1554854 - 1555790] |
| slr2112 | 1.418 | 0.000 | 1.342 | 0.000 | putative transposase [ISY100o: 1626093 - 1627038] |
| sll0661 | 1.411 | 0.000 | 1.477 | 0.000 | hypothetical protein |
| sll5130 | 1.409 | 0.001 | 1.137 | 0.009 | hypothetical protein |
| sll0810 | 1.409 | 0.000 | 1.644 | 0.000 | unknown protein |
| slr1613 | 1.408 | 0.000 | 1.356 | 0.000 | hypothetical protein |
| sll1225 | 1.407 | 0.000 | 0.969 | 0.000 | unknown protein |
| slr1236 | 1.404 | 0.000 | 1.343 | 0.000 | hypothetical protein |
| slr1522 | 1.403 | 0.000 | 1.077 | 0.000 | putative transposase [ISY352d: 1614422 - 1615835] |
| ssl7019 | 1.402 | 0.000 | 1.889 | 0.000 | unknown protein |
| sll0178 | 1.392 | 0.000 | 1.343 | 0.000 | hypothetical protein |
| slr6049 | 1.380 | 0.000 | 1.167 | 0.000 | hypothetical protein |
| sll1297 | 1.370 | 0.000 | 1.481 | 0.000 | probable dioxygenase, Rieske iron-sulfur component |
| sll1200 | 1.363 | 0.000 | 1.276 | 0.000 | hypothetical protein |
| sll0650 | 1.347 | 0.000 | 1.320 | 0.000 | putative transposase [ISY100j: 421739 - 422684] |
| sll5002 | 1.345 | 0.000 | 1.213 | 0.002 | hypothetical protein |
| sll0101 | 1.334 | 0.000 | 1.480 | 0.000 | unknown protein |
| slr1614 | 1.329 | 0.000 | 1.087 | 0.000 | hypothetical protein |
| sll0687 | 1.328 | 0.000 | 0.997 | 0.000 | RNA polymerase ECF-type (group 3) sigma factor |
| slr1490 | 1.326 | 0.000 | 1.420 | 0.000 | ferrichrome-iron receptor |
| slr5010 | 1.326 | 0.000 | 1.407 | 0.000 | integrase/recombinase |
| slr1033 | 1.321 | 0.000 | 1.169 | 0.000 | unknown protein |

| | | | | | |
|---|---|---|---|---|---|
| sll1164 | 1.321 | 0.000 | 1.176 | 0.000 | hypothetical protein |
| slr1616 | 1.320 | 0.000 | 1.362 | 0.000 | unknown protein |
| sll1999 | 1.318 | 0.000 | 1.277 | 0.000 | putative transposase [ISY203h: 1623060 - 1623693, join 1624643 - 1625182] |
| slr0179 | 1.317 | 0.000 | 1.348 | 0.000 | hypothetical protein |
| slr1615 | 1.315 | 0.000 | 1.166 | 0.000 | perosamine synthetase |
| sll1240 | 1.314 | 0.000 | 1.425 | 0.000 | unknown protein |
| slr5124 | 1.312 | 0.000 | 1.148 | 0.000 | hypothetical protein |
| sll1985 | 1.309 | 0.000 | 1.218 | 0.000 | putative transposase [ISY352c: 1553414 - 1553903, join 1554854 - 1555790] |
| ssl1263 | 1.306 | 0.000 | 1.466 | 0.000 | hypothetical protein |
| slr5127 | 1.305 | 0.000 | 1.404 | 0.000 | unknown protein |
| sll0200 | 1.300 | 0.000 | 1.290 | 0.000 | putative transposase [ISY100s: 2524547 - 2525492] |
| sll1983 | 1.299 | 0.000 | 1.284 | 0.000 | putative transposase [ISY100n: 1553903 - 1554848] |
| slr0982 | 1.299 | 0.000 | 1.276 | 0.000 | probable polysaccharide ABC transporter ATP binding subunit |
| slr7104 | 1.297 | 0.000 | 1.286 | 0.000 | putative transposase [ISY100w: 100709 - 101654] |
| sll1256 | 1.296 | 0.000 | 1.291 | 0.000 | putative transposase [ISY100p: 1725405 - 1726350] |
| slr0727 | 1.294 | 0.000 | 1.289 | 0.000 | unknown protein |
| slr1936 | 1.294 | 0.000 | 1.285 | 0.000 | putative transposase [ISY100r: 2235489 - 2236434] |
| slr0106 | 1.294 | 0.000 | 1.092 | 0.000 | unknown protein |
| sll1436 | 1.294 | 0.000 | 1.288 | 0.000 | putative transposase [ISY100q: 1901359 - 1902304] |
| sll1880 | 1.294 | 0.000 | 1.349 | 0.000 | hypothetical protein |
| sll0909 | 1.293 | 0.000 | 1.259 | 0.000 | unknown protein |
| sll0699 | 1.293 | 0.000 | 1.287 | 0.000 | putative transposase [ISY100i: 123475 - 124420] |
| ssl5025 | 1.292 | 0.005 | 1.525 | 0.001 | hypothetical protein |

| | | | | | |
|---|---|---|---|---|---|
| slr1715 | 1.290 | 0.000 | 1.285 | 0.000 | putative transposase [ISY100m: 1463383 - 1464328] |
| sll0658 | 1.277 | 0.000 | 1.250 | 0.000 | hypothetical protein |
| slr1930 | 1.268 | 0.000 | 1.276 | 0.000 | hypothetical protein |
| slr1118 | 1.261 | 0.000 | 1.199 | 0.000 | probable UDP-N-acetyl-D-mannosaminuronic acid transferase |
| sll1472 | 1.259 | 0.003 | 1.127 | 0.014 | unknown protein |
| ssl5070 | 1.247 | 0.003 | 1.475 | 0.000 | unknown protein |
| slr1045 | 1.244 | 0.000 | 1.152 | 0.000 | hypothetical protein YCF63 |
| slr1524 | 1.242 | 0.000 | 1.234 | 0.000 | putative transposase [ISY100u(partial copy): 1616832 - 1617509] |
| sll5083 | 1.235 | 0.000 | 1.124 | 0.000 | unknown protein |
| sll0662 | 1.235 | 0.000 | 1.268 | 0.000 | ferredoxin |
| sll0384 | 1.229 | 0.000 | 1.111 | 0.000 | unknown protein |
| slr2083 | 1.229 | 0.000 | 1.051 | 0.000 | cytochrome c oxidase subunit III |
| ssl2559 | 1.229 | 0.000 | 0.996 | 0.002 | ferredoxin |
| sll0382 | 1.229 | 0.000 | 1.138 | 0.000 | hypothetical protein |
| slr0468 | 1.228 | 0.000 | 1.272 | 0.000 | unknown protein |
| ssr1473 | 1.227 | 0.001 | 1.368 | 0.000 | hypothetical protein |
| sll5063 | 1.224 | 0.000 | 1.247 | 0.000 | hypothetical protein |
| slr1048 | 1.222 | 0.000 | 1.169 | 0.000 | hypothetical protein |
| sll1304 | 1.221 | 0.000 | 1.065 | 0.000 | hypothetical protein |
| slr1864 | 1.219 | 0.000 | 1.282 | 0.000 | hypothetical protein |
| sll0666 | 1.216 | 0.000 | 1.057 | 0.001 | putative transposase [ISY523r: 3109761 - 3110626] |
| slr0984 | 1.216 | 0.000 | 1.191 | 0.000 | CDP-glucose 4,6-dehydratase |
| slr0852 | 1.213 | 0.000 | 1.351 | 0.000 | hypothetical protein |
| ssr2912 | 1.210 | 0.000 | 1.070 | 0.001 | unknown protein |
| slr5073 | 1.206 | 0.000 | 1.042 | 0.000 | unknown protein |

| | | | | | |
|---|---|---|---|---|---|
| slr2007 | 1.199 | 0.000 | 1.292 | 0.000 | NADH dehydrogenase subunit 4 |
| slr0977 | 1.192 | 0.000 | 1.097 | 0.000 | ABC transporter, permease component |
| slr1493 | 1.188 | 0.000 | 1.060 | 0.001 | hypothetical protein |
| slr1075 | 1.187 | 0.000 | 1.116 | 0.000 | putative transposase [ISY100b: 378993 - 379939] |
| ssl0426 | 1.185 | 0.000 | 1.216 | 0.000 | putative transposase [ISY100t(partial copy): 141097 - 141410] |
| sll0381 | 1.183 | 0.000 | 0.955 | 0.000 | hypothetical protein |
| ssr1038 | 1.182 | 0.000 | 1.032 | 0.000 | unknown protein |
| sll1241 | 1.180 | 0.000 | 1.121 | 0.000 | unknown protein |
| slr0286 | 1.180 | 0.000 | 1.086 | 0.000 | protein involved in functional assembly of photosystem II |
| slr0870 | 1.176 | 0.000 | 1.186 | 0.000 | hypothetical protein |
| slr7073 | 1.174 | 0.000 | 1.206 | 0.000 | hypothetical protein |
| slr0853 | 1.174 | 0.000 | 1.353 | 0.000 | ribosomal-protein-alanine acetyltransferase |
| ssr2201 | 1.174 | 0.000 | 1.382 | 0.000 | unknown protein |
| sll1687 | 1.172 | 0.000 | 1.136 | 0.000 | unknown protein |
| slr1980 | 1.170 | 0.000 | 1.061 | 0.000 | unknown protein |
| ssr1175 | 1.169 | 0.000 | 1.180 | 0.000 | putative transposase [ISY100v: 3095975 - 3096319, join 3097194 - 3097362, join 3098314 - 3098743] |
| slr1305 | 1.166 | 0.000 | 1.175 | 0.000 | two-component response regulator |
| slr5078 | 1.164 | 0.000 | 1.197 | 0.000 | similar to potassium channel protein |
| ssr3467 | 1.157 | 0.001 | 1.015 | 0.003 | unknown protein |
| ssr2899 | 1.155 | 0.003 | 1.084 | 0.006 | putative transposase [ISY523m(partial copy): 1483390 - 1484062] |
| slr0231 | 1.155 | 0.013 | 1.138 | 0.007 | probable DNA-3-methyladenine glycosylase |
| slr7024 | 1.147 | 0.000 | 1.125 | 0.000 | hypothetical protein |
| sll5079 | 1.142 | 0.000 | 1.135 | 0.000 | probable short chain dehydrogenase |
| slr1209 | 1.139 | 0.000 | 1.027 | 0.000 | hypothetical protein |

| | | | | | |
|---|---|---|---|---|---|
| sll0385 | 1.139 | 0.000 | 1.100 | 0.000 | ATP-binding protein of ABC transporter |
| slr1357 | 1.138 | 0.000 | 1.043 | 0.000 | putative transposase [ISY100c: 1098251 - 1099197] |
| slr7080 | 1.133 | 0.000 | 1.066 | 0.000 | unknown protein |
| ssl2874 | 1.131 | 0.000 | 1.145 | 0.000 | hypothetical protein |
| sll0449 | 1.128 | 0.000 | 1.204 | 0.000 | unknown protein |
| ssl7074 | 1.128 | 0.000 | 1.131 | 0.000 | hypothetical protein |
| slr1417 | 1.127 | 0.000 | 1.267 | 0.000 | hypothetical protein YCF57 |
| slr0976 | 1.127 | 0.000 | 1.176 | 0.000 | hypothetical protein |
| slr0300 | 1.123 | 0.000 | 1.146 | 0.000 | hypothetical protein |
| slr7026 | 1.122 | 0.017 | 1.358 | 0.002 | unknown protein |
| slr0914 | 1.122 | 0.000 | 1.033 | 0.000 | unknown protein |
| slr5082 | 1.118 | 0.000 | 1.001 | 0.000 | hypothetical protein |
| slr1612 | 1.116 | 0.000 | 1.076 | 0.000 | hypothetical protein |
| sll5014 | 1.115 | 0.008 | 1.086 | 0.010 | similar to maturase |
| sll1642 | 1.114 | 0.003 | 0.766 | 0.081 | hypothetical protein |
| sll5132 | 1.114 | 0.000 | 1.100 | 0.000 | hypothetical protein |
| ssr7018 | 1.111 | 0.013 | 1.495 | 0.001 | unknown protein |
| ssl1255 | 1.111 | 0.000 | 1.075 | 0.000 | hypothetical protein |
| sll1998 | 1.110 | 0.000 | 1.028 | 0.000 | putative transposase [ISY100d: 1623697 - 1624643] |
| ssr3129 | 1.109 | 0.000 | 1.042 | 0.000 | unknown protein |
| sll1061 | 1.108 | 0.000 | 0.850 | 0.000 | unknown protein |
| slr0675 | 1.107 | 0.000 | 1.175 | 0.000 | unknown protein |
| sll0241 | 1.106 | 0.000 | 1.137 | 0.000 | unknown protein |
| slr0230 | 1.104 | 0.000 | 0.965 | 0.000 | putative transposase [ISY100f: 2534034 - 2534980] |
| slr2036 | 1.103 | 0.000 | 1.105 | 0.000 | putative transposase [ISY203a: 573408 - 574580] |

| | | | | | |
|---|---|---|---|---|---|
| sll0375 | 1.100 | 0.000 | 1.139 | 0.000 | unknown protein |
| slr0755 | 1.097 | 0.000 | 0.945 | 0.000 | hypothetical protein |
| sll1397 | 1.096 | 0.000 | 1.007 | 0.000 | putative transposase [ISY100a: 52234 - 53180] |
| slr0704 | 1.094 | 0.000 | 0.961 | 0.000 | putative transposase [ISY100g: 3097363 - 3098309] |
| sll1439 | 1.094 | 0.009 | 1.023 | 0.015 | unknown protein |
| slr1046 | 1.093 | 0.000 | 0.970 | 0.000 | putative TatA protein |
| slr5054 | 1.092 | 0.000 | 1.002 | 0.000 | glycosyl transferase |
| sll1305 | 1.092 | 0.000 | 1.042 | 0.000 | hypothetical protein |
| sll0651 | 1.091 | 0.000 | 0.943 | 0.000 | putative transposase [ISY100j: 421739 - 422684] |
| sll0431 | 1.091 | 0.000 | 1.001 | 0.000 | putative transposase [ISY100h: 3512289 - 3513235] |
| slr5118 | 1.082 | 0.000 | 0.827 | 0.000 | hypothetical protein |
| sll5128 | 1.079 | 0.000 | 1.068 | 0.000 | unknown protein |
| ssl7004 | 1.076 | 0.001 | 1.257 | 0.000 | probable plasmid stability protein |
| sll0092 | 1.075 | 0.000 | 0.953 | 0.000 | putative transposase [ISY391c: 2997600 - 2998989] |
| slr1857 | 1.074 | 0.000 | 0.988 | 0.000 | isoamylase |
| slr2077 | 1.073 | 0.000 | 1.082 | 0.000 | probable ABC transporter, periplasmic binding protein |
| slr0180 | 1.072 | 0.000 | 1.170 | 0.000 | putative transposase [ISY203f: 2326926 - 2328099] |
| slr1789 | 1.072 | 0.000 | 1.302 | 0.000 | unknown protein |
| sll1232 | 1.070 | 0.000 | 1.016 | 0.000 | hypothetical protein |
| slr0352 | 1.066 | 0.000 | 1.002 | 0.000 | putative transposase [ISY100e: 2443927 - 2444873] |
| slr1992 | 1.063 | 0.000 | 1.083 | 0.000 | glutathione peroxidase-like NADPH peroxidase |
| ssl2921 | 1.061 | 0.000 | 1.167 | 0.000 | hypothetical protein |
| slr5005 | 1.061 | 0.000 | 1.051 | 0.000 | hypothetical protein |
| ssr2062 | 1.056 | 0.000 | 1.061 | 0.000 | hypothetical protein |
| slr0407 | 1.055 | 0.000 | 0.944 | 0.000 | hypothetical protein |

| | | | | | |
|---|---|---|---|---|---|
| slr5102 | 1.054 | 0.002 | 1.100 | 0.004 | hypothetical protein |
| slr0323 | 1.053 | 0.000 | 1.098 | 0.000 | putative alpha-mannosidase |
| slr1690 | 1.053 | 0.000 | 0.861 | 0.001 | hypothetical protein |
| sll1930 | 1.051 | 0.000 | 0.891 | 0.000 | putative transposase [ISY100k: 605515 - 606460] |
| slr1403 | 1.050 | 0.000 | 1.044 | 0.000 | unknown protein |
| slr2010 | 1.047 | 0.000 | 0.989 | 0.000 | hypothetical protein |
| slr0311 | 1.041 | 0.000 | 0.922 | 0.000 | two-component sensor histidine kinase |
| sll7069 | 1.041 | 0.000 | 0.993 | 0.000 | hypothetical protein |
| slr1584 | 1.040 | 0.000 | 0.933 | 0.000 | two-component transcription regulator OmpR subfamily |
| slr0092 | 1.039 | 0.000 | 0.813 | 0.001 | hypothetical protein |
| ssl7039 | 1.039 | 0.000 | 1.154 | 0.000 | hypothetical protein |
| sll1307 | 1.038 | 0.000 | 1.050 | 0.000 | periplasmic protein, function unknown |
| sll1231 | 1.035 | 0.000 | 1.005 | 0.000 | mannosyltransferase |
| ssl3382 | 1.034 | 0.000 | 1.047 | 0.000 | hypothetical protein |
| slr1047 | 1.033 | 0.000 | 1.136 | 0.000 | hypothetical protein |
| ssl1046 | 1.033 | 0.000 | 1.105 | 0.000 | hypothetical protein |
| slr1393 | 1.033 | 0.000 | 0.921 | 0.000 | phytochrome-like protein, two-component sensor histidine kinase |
| sll1780 | 1.029 | 0.000 | 1.107 | 0.000 | putative transposase [ISY203b: 1200306 - 1201479] |
| slr1635 | 1.029 | 0.000 | 1.107 | 0.000 | putative transposase [ISY203e: 2048410 - 2049583] |
| sll1474 | 1.029 | 0.000 | 1.107 | 0.000 | putative transposase [ISY203g: 3400332 - 3401505] |
| sll5131 | 1.029 | 0.000 | 1.107 | 0.000 | putative transposase [ISY203j: 117275 - 118448] |
| slr6105 | 1.029 | 0.000 | 1.107 | 0.000 | putative transposase [ISY203x: 99448 - 100621] |
| sll0265 | 1.026 | 0.000 | 1.157 | 0.000 | unknown protein |
| slr1025 | 1.025 | 0.001 | 1.029 | 0.000 | hypothetical protein |
| sll7070 | 1.025 | 0.000 | 1.217 | 0.000 | unknown protein |

| | | | | | |
|---|---|---|---|---|---|
| sll5081 | 1.022 | 0.027 | 1.220 | 0.006 | hypothetical protein |
| sll1161 | 1.020 | 0.000 | 0.994 | 0.000 | probable adenylate cyclase |
| sll1447 | 1.013 | 0.000 | 0.933 | 0.001 | hypothetical protein |
| slr0857 | 1.010 | 0.000 | 0.778 | 0.000 | putative transposase [ISY100l: 1346125 - 1347070] |
| sll0723 | 1.009 | 0.000 | 0.986 | 0.000 | hypothetical protein |
| sll1233 | 1.006 | 0.000 | 1.002 | 0.000 | hypothetical protein |
| sll0168 | 1.006 | 0.000 | 0.729 | 0.002 | hypothetical protein |
| slr0294 | 1.004 | 0.000 | 0.868 | 0.001 | unknown protein |
| sll1003 | 1.000 | 0.000 | 1.001 | 0.000 | two-component sensor histidine kinase |
| sll0264 | 0.994 | 0.000 | 1.064 | 0.000 | probable dioxygenase Rieske iron-sulfur component |
| ssr0757 | 0.988 | 0.000 | 1.207 | 0.000 | hypothetical protein |
| sll1582 | 0.984 | 0.000 | 1.029 | 0.000 | unknown protein |
| slr7008 | 0.983 | 0.000 | 1.084 | 0.000 | putative transposase [ISY203k: 4154 - 5327] |
| sll0024 | 0.978 | 0.000 | 1.081 | 0.000 | unknown protein |
| sll1672 | 0.974 | 0.000 | 1.035 | 0.000 | two-component hybrid sensor and regulator |
| slr0517 | 0.973 | 0.000 | 1.016 | 0.000 | hypothetical protein |
| sll1997 | 0.952 | 0.000 | 1.148 | 0.000 | putative transposase [ISY203h: 1623060 - 1623693, join 1624643 - 1625182] |
| slr8036 | 0.949 | 0.000 | 1.011 | 0.000 | probable acetyltransferase |
| sll1596 | 0.941 | 0.001 | 1.069 | 0.000 | circadian clock protein KaiB homolog |
| slr0856 | 0.941 | 0.000 | 1.084 | 0.000 | putative transposase [ISY100l: 1346125 - 1347070] |
| sll1598 | 0.932 | 0.000 | 1.029 | 0.000 | metal ABC transporter substrate-binding protein |
| ssr0755 | 0.904 | 0.000 | 1.017 | 0.000 | hypothetical protein |
| slr0273 | 0.903 | 0.000 | 1.007 | 0.000 | unknown protein |
| slr0790 | 0.898 | 0.015 | 1.110 | 0.003 | similar to ultraviolet light resistance protein B |

| | | | | | |
|---|---|---|---|---|---|
| slr1162 | 0.877 | 0.000 | 1.052 | 0.000 | unknown protein |
| ssl3364 | 0.863 | 0.000 | 1.045 | 0.000 | CP12 polypeptide |
| slr7096 | 0.833 | 0.003 | 1.155 | 0.000 | hypothetical protein |
| ssr2848 | 0.833 | 0.000 | 1.085 | 0.000 | unknown protein |
| sll1476 | 0.824 | 0.026 | 1.112 | 0.001 | unknown protein |
| ssr0706 | 0.779 | 0.004 | 1.052 | 0.000 | unknown protein |
| ssl8028 | 0.708 | 0.001 | 1.000 | 0.000 | hypothetical protein |
| slr0573 | 0.642 | 0.212 | 1.266 | 0.002 | unknown protein |
| sll0268 | -0.731 | 0.000 | -1.018 | 0.000 | hypothetical protein |
| sll1386 | -0.790 | 0.001 | -1.131 | 0.000 | hypothetical protein |
| slr1462 | -0.838 | 0.000 | -1.036 | 0.000 | hypothetical protein |
| ssr2549 | -0.845 | 0.002 | -1.110 | 0.000 | unknown protein |
| sll0419 | -0.855 | 0.002 | -1.095 | 0.000 | unknown protein |
| slr1418 | -0.885 | 0.000 | -1.036 | 0.000 | dihydroorotate dehydrogenase |
| slr0448 | -0.895 | 0.000 | -1.144 | 0.000 | DNA repair protein RadA |
| sml0006 | -0.907 | 0.000 | -1.024 | 0.000 | 50S ribosomal protein L36 |
| slr0592 | -0.918 | 0.000 | -1.105 | 0.000 | hypothetical protein |
| slr1431 | -0.919 | 0.000 | -1.017 | 0.000 | hypothetical protein |
| sll1081 | -0.919 | 0.000 | -1.095 | 0.000 | ABC transporter permease |
| slr1913 | -0.930 | 0.001 | -1.167 | 0.000 | hypothetical protein |
| sll1755 | -0.942 | 0.000 | -1.031 | 0.000 | unknown protein |
| sll0933 | -0.967 | 0.000 | -1.004 | 0.000 | hypothetical protein |
| slr2107 | -0.969 | 0.019 | -1.638 | 0.000 | probable polysaccharide ABC transporter permease protein |
| slr1875 | -0.979 | 0.000 | -1.049 | 0.000 | hypothetical protein |
| ssl1417 | -0.989 | 0.001 | -1.194 | 0.000 | hypothetical protein YCF33 |

| | | | | | |
|---|---|---|---|---|---|
| sll0708 | -0.996 | 0.005 | -1.386 | 0.000 | dimethyladenosine transferase |
| slr6006 | -0.996 | 0.005 | -1.184 | 0.001 | unknown protein |
| slr6065 | -0.996 | 0.005 | -1.184 | 0.001 | unknown protein |
| slr0708 | -0.998 | 0.000 | -1.013 | 0.000 | periplasmic protein, function unknown |
| slr1963 | -1.002 | 0.000 | -0.839 | 0.000 | water-soluble carotenoid protein |
| sll0252 | -1.004 | 0.000 | -0.910 | 0.000 | unknown protein |
| sll1817 | -1.004 | 0.000 | -1.111 | 0.000 | 30S ribosomal protein S11 |
| slr0929 | -1.007 | 0.000 | -1.006 | 0.000 | chromosome partitioning protein, ParA family |
| slr0744 | -1.008 | 0.000 | -1.047 | 0.000 | translation initiation factor IF-2 |
| sll1822 | -1.008 | 0.000 | -0.832 | 0.000 | 30S ribosomal protein S9 |
| sll1181 | -1.012 | 0.000 | -0.926 | 0.000 | similar to hemolysin secretion protein |
| slr1734 | -1.013 | 0.000 | -1.003 | 0.000 | glucose 6-phosphate dehydrogenase assembly protein |
| ssl3342 | -1.013 | 0.000 | -0.796 | 0.002 | hypothetical protein |
| slr1471 | -1.019 | 0.000 | -0.965 | 0.000 | hypothetical protein |
| slr1886 | -1.019 | 0.000 | -0.943 | 0.000 | hypothetical protein |
| slr1906 | -1.021 | 0.000 | -1.016 | 0.000 | hypothetical protein |
| sll0421 | -1.023 | 0.000 | -0.766 | 0.000 | adenylosuccinate lyase |
| slr1670 | -1.025 | 0.000 | -1.034 | 0.000 | unknown protein |
| sll1491 | -1.027 | 0.000 | -1.134 | 0.000 | periplasmic WD-repeat protein |
| slr0417 | -1.028 | 0.000 | -1.127 | 0.000 | DNA gyrase subunit A |
| sll1886 | -1.029 | 0.000 | -1.020 | 0.000 | hypothetical protein |
| slr0291 | -1.029 | 0.000 | -1.246 | 0.000 | hypothetical protein |
| slr1096 | -1.032 | 0.000 | -1.119 | 0.000 | dihydrolipoamide dehydrogenase |
| sll1079 | -1.034 | 0.000 | -1.155 | 0.000 | hydrogenase accessory protein HypB |
| sll1184 | -1.037 | 0.000 | -0.807 | 0.000 | heme oxygenase |

| | | | | | |
|---|---|---|---|---|---|
| slr0924 | -1.037 | 0.000 | -1.036 | 0.000 | periplasmic protein, function unknown |
| sll1623 | -1.039 | 0.000 | -0.787 | 0.000 | ABC transporter ATP-binding protein |
| slr1791 | -1.040 | 0.000 | -1.055 | 0.000 | phosphoadenosine phosphosulfate reductase |
| slr0083 | -1.043 | 0.000 | -0.892 | 0.000 | RNA helicase Light |
| ssr2595 | -1.044 | 0.000 | -0.724 | 0.000 | high light-inducible polypeptide HliB, CAB/ELIP/HLIP superfamily |
| sll1892 | -1.044 | 0.000 | -1.023 | 0.000 | unknown protein |
| slr0954 | -1.046 | 0.000 | -1.106 | 0.000 | hypothetical protein |
| slr0797 | -1.047 | 0.000 | -1.059 | 0.000 | cobalt-transporting P-type ATPase (cobalt efflux pump) involved in cobalt tolerance |
| sll1377 | -1.048 | 0.000 | -1.050 | 0.000 | probable glycosyltransferase |
| slr0657 | -1.050 | 0.000 | -0.984 | 0.000 | aspartate kinase |
| slr0146 | -1.051 | 0.000 | -1.028 | 0.000 | hypothetical protein |
| sll1236 | -1.052 | 0.000 | -1.148 | 0.000 | unknown protein |
| sll1823 | -1.053 | 0.000 | -1.008 | 0.000 | adenylosuccinate synthetase |
| slr1160 | -1.056 | 0.000 | -0.857 | 0.000 | periplasmic protein, function unknown |
| slr1544 | -1.058 | 0.000 | -0.823 | 0.000 | unknown protein |
| slr1885 | -1.059 | 0.000 | -1.169 | 0.000 | hypothetical protein |
| slr1390 | -1.061 | 0.000 | -1.046 | 0.000 | cell division protein FtsH |
| sll0767 | -1.061 | 0.000 | -0.944 | 0.000 | 50S ribosomal protein L20 |
| slr0469 | -1.064 | 0.000 | -1.083 | 0.000 | 30S ribosomal protein S4 |
| slr0397 | -1.066 | 0.000 | -1.166 | 0.000 | hypothetical protein |
| sll1274 | -1.068 | 0.000 | -0.739 | 0.000 | hypothetical protein |
| slr0112 | -1.070 | 0.000 | -1.204 | 0.000 | unknown protein |
| slr1946 | -1.073 | 0.000 | -1.103 | 0.000 | hypothetical protein |
| slr1708 | -1.078 | 0.000 | -0.992 | 0.000 | probable peptidase |

| | | | | | |
|---|---|---|---|---|---|
| slr1898 | -1.081 | 0.000 | -1.082 | 0.000 | N-acetylglutamate kinase |
| slr0368 | -1.083 | 0.000 | -0.958 | 0.000 | unknown protein |
| slr6022 | -1.086 | 0.001 | -1.595 | 0.000 | unknown protein |
| slr6081 | -1.086 | 0.001 | -1.595 | 0.000 | unknown protein |
| slr0088 | -1.088 | 0.000 | -0.841 | 0.002 | beta-carotene ketolase |
| sll1980 | -1.090 | 0.000 | -0.778 | 0.000 | thiol:disulfide interchange protein TrxA |
| slr1949 | -1.090 | 0.000 | -0.884 | 0.000 | hypothetical protein |
| ssr1736 | -1.090 | 0.000 | -0.880 | 0.000 | 50S ribosomal protein L32 |
| slr1260 | -1.091 | 0.000 | -1.014 | 0.000 | hypothetical protein |
| sll1049 | -1.098 | 0.000 | -1.073 | 0.000 | hypothetical protein |
| sll1818 | -1.098 | 0.000 | -1.055 | 0.000 | RNA polymerase alpha subunit |
| sll1130 | -1.100 | 0.000 | -1.045 | 0.000 | unknown protein |
| sll1142 | -1.101 | 0.000 | -1.071 | 0.000 | hypothetical protein |
| sll1077 | -1.104 | 0.000 | -1.081 | 0.000 | agmatinase |
| sll1874 | -1.104 | 0.001 | -1.178 | 0.000 | magnesium-protoporphyrin IX monomethyl ester (oxidative) cyclase |
| ssr2142 | -1.106 | 0.000 | -0.831 | 0.000 | hypothetical protein YCF19 |
| ssr2554 | -1.108 | 0.000 | -0.874 | 0.000 | hypothetical protein |
| ssl2501 | -1.109 | 0.000 | -0.859 | 0.000 | unknown protein |
| sll8034 | -1.110 | 0.002 | -0.669 | 0.062 | 2-nitropropane dioxygenase |
| slr0585 | -1.111 | 0.000 | -1.044 | 0.000 | argininosuccinate synthetase |
| sll1096 | -1.111 | 0.000 | -1.030 | 0.000 | 30S ribosomal protein S12 |
| ssr2194 | -1.114 | 0.000 | -1.307 | 0.000 | hypothetical protein |
| sll1832 | -1.115 | 0.000 | -0.996 | 0.000 | hypothetical protein |
| sll1029 | -1.116 | 0.000 | -1.107 | 0.000 | carbon dioxide concentrating mechanism protein CcmK |
| slr0665 | -1.123 | 0.000 | -1.038 | 0.000 | aconitate hydratase |

| | | | | | |
|---|---|---|---|---|---|
| slr1187 | -1.127 | 0.000 | -1.031 | 0.000 | unknown protein |
| slr1201 | -1.130 | 0.000 | -1.188 | 0.001 | urea transport system permease protein |
| sll0469 | -1.130 | 0.000 | -1.030 | 0.000 | ribose-phosphate pyrophosphokinase |
| sll1185 | -1.132 | 0.000 | -1.162 | 0.000 | coproporphyrinogen III oxidase, aerobic (oxygen-dependent) |
| slr0963 | -1.140 | 0.000 | -1.156 | 0.000 | ferredoxin-sulfite reductase |
| sll1949 | -1.142 | 0.000 | -1.136 | 0.000 | unknown protein |
| sll1330 | -1.144 | 0.000 | -1.140 | 0.000 | two-component system response regulator OmpR subfamily |
| sll0173 | -1.144 | 0.000 | -1.000 | 0.000 | virginiamycin B hydrolase, periplasmic protein |
| slr1302 | -1.144 | 0.000 | -1.196 | 0.000 | protein involved in constitutive low affinity CO2 uptake |
| slr1289 | -1.152 | 0.000 | -1.112 | 0.000 | isocitrate dehydrogenase (NADP+) |
| sll0208 | -1.162 | 0.000 | -1.029 | 0.000 | hypothetical protein |
| ssl2148 | -1.163 | 0.000 | -1.081 | 0.000 | hypothetical protein |
| slr1909 | -1.171 | 0.000 | -1.294 | 0.000 | two-component response regulator NarL subfamily |
| sll0716 | -1.171 | 0.000 | -1.226 | 0.000 | leader peptidase I (signal peptidase I) |
| sll1837 | -1.176 | 0.000 | -1.423 | 0.000 | hypothetical protein |
| slr2136 | -1.180 | 0.000 | -1.022 | 0.000 | GcpE protein homolog |
| sll1931 | -1.181 | 0.000 | -0.990 | 0.000 | serine hydroxymethyltransferase |
| sll0623 | -1.186 | 0.000 | -1.127 | 0.000 | unknown protein |
| slr0287 | -1.187 | 0.000 | -1.262 | 0.000 | hypothetical protein |
| ssl3769 | -1.188 | 0.000 | -1.061 | 0.000 | unknown protein |
| sll1172 | -1.189 | 0.000 | -1.097 | 0.000 | threonine synthase |
| slr1577 | -1.190 | 0.003 | -1.079 | 0.009 | hypothetical protein |
| slr0006 | -1.194 | 0.000 | -1.329 | 0.000 | unknown protein |
| sll1942 | -1.195 | 0.000 | -1.145 | 0.000 | unknown protein |
| sll0293 | -1.197 | 0.000 | -1.170 | 0.000 | unknown protein |

| | | | | | |
|---|---|---|---|---|---|
| slr1838 | -1.198 | 0.000 | -1.061 | 0.000 | carbon dioxide concentrating mechanism protein CcmK homolog 3, putative carboxysome assembly protein |
| slr1202 | -1.198 | 0.000 | -1.193 | 0.000 | permease protein of sugar ABC transporter |
| slr2067 | -1.205 | 0.000 | -1.107 | 0.000 | allophycocyanin alpha subunit |
| sll1740 | -1.205 | 0.000 | -1.033 | 0.000 | 50S ribosomal protein L19 |
| slr0009 | -1.208 | 0.000 | -1.078 | 0.000 | ribulose bisphosphate carboxylase large subunit |
| sll1744 | -1.210 | 0.000 | -1.294 | 0.000 | 50S ribosomal protein L1 |
| sll1816 | -1.211 | 0.000 | -1.289 | 0.000 | 30S ribosomal protein S13 |
| sll1743 | -1.212 | 0.000 | -1.290 | 0.000 | 50S ribosomal protein L11 |
| slr1964 | -1.219 | 0.000 | -1.113 | 0.000 | hypothetical protein |
| sll0755 | -1.220 | 0.000 | -1.254 | 0.000 | thioredoxin peroxidase |
| slr0011 | -1.221 | 0.000 | -1.150 | 0.000 | possible Rubisco chaperonin |
| sll1621 | -1.222 | 0.000 | -1.094 | 0.000 | AhpC/TSA family protein |
| slr1535 | -1.224 | 0.000 | -1.131 | 0.000 | hypothetical protein |
| slr2127 | -1.226 | 0.000 | -1.044 | 0.000 | hypothetical protein |
| sll1338 | -1.228 | 0.000 | -1.103 | 0.000 | unknown protein |
| slr0734 | -1.233 | 0.000 | -1.199 | 0.000 | hypothetical protein |
| slr0748 | -1.239 | 0.000 | -1.194 | 0.000 | hypothetical protein |
| sll0418 | -1.240 | 0.000 | -1.194 | 0.000 | 2-methyl-6-phytylbenzoquinone methyltransferase |
| slr1673 | -1.242 | 0.000 | -1.020 | 0.000 | probable tRNA/rRNA methyltransferase |
| slr1505 | -1.244 | 0.000 | -1.166 | 0.000 | unknown protein |
| sll0007 | -1.244 | 0.000 | -1.033 | 0.000 | hypothetical protein |
| sll1483 | -1.244 | 0.000 | -1.122 | 0.000 | periplasmic protein, similar to transforming growth factor induced protein |
| sll1340 | -1.249 | 0.009 | -1.818 | 0.001 | hypothetical protein |
| sll1724 | -1.251 | 0.000 | -1.396 | 0.000 | glycosyl transferase family 1 |

| | | | | | |
|---|---|---|---|---|---|
| sll1584 | -1.252 | 0.000 | -1.180 | 0.000 | ferredoxin like protein |
| sll0199 | -1.253 | 0.000 | -1.028 | 0.000 | plastocyanin |
| slr1549 | -1.257 | 0.000 | -1.165 | 0.000 | polypeptide deformylase |
| sll1819 | -1.258 | 0.000 | -1.139 | 0.000 | 50S ribosomal protein L17 |
| slr0994 | -1.264 | 0.000 | -1.201 | 0.000 | lipoate-protein ligase B |
| slr1170 | -1.265 | 0.000 | -1.184 | 0.001 | hypothetical protein |
| slr0366 | -1.265 | 0.000 | -1.133 | 0.000 | unknown protein |
| slr0753 | -1.267 | 0.000 | -1.218 | 0.000 | probable transport protein |
| slr6095 | -1.274 | 0.000 | -1.101 | 0.002 | type I restriction-modification system, M subunit (fragment) |
| slr6005 | -1.276 | 0.007 | -1.342 | 0.009 | unknown protein |
| slr6064 | -1.276 | 0.007 | -1.342 | 0.009 | unknown protein |
| sll1815 | -1.276 | 0.000 | -1.355 | 0.000 | adenylate kinase |
| slr0955 | -1.276 | 0.000 | -1.295 | 0.000 | probable tRNA/rRNA methyltransferase |
| sll1799 | -1.277 | 0.000 | -1.312 | 0.000 | 50S ribosomal protein L3 |
| sll1123 | -1.280 | 0.000 | -1.279 | 0.000 | hypothetical protein |
| slr0505 | -1.286 | 0.000 | -1.211 | 0.000 | hypothetical protein |
| sll6054 | -1.289 | 0.000 | -0.849 | 0.001 | hypothetical protein |
| sll1746 | -1.292 | 0.000 | -1.269 | 0.000 | 50S ribosomal protein L12 |
| sll0757 | -1.302 | 0.000 | -1.194 | 0.000 | amidophosphoribosyltransferase |
| sll0373 | -1.304 | 0.000 | -1.317 | 0.000 | gamma-glutamyl phosphate reductase |
| slr0798 | -1.307 | 0.000 | -1.415 | 0.000 | ATPase P |
| sll1885 | -1.327 | 0.000 | -1.294 | 0.000 | unknown protein |
| sll0312 | -1.340 | 0.000 | -1.404 | 0.000 | probable oligopeptides ABC transporter permease protein |
| slr1469 | -1.341 | 0.000 | -1.419 | 0.000 | protein subunit of ribonuclease P (RNase P) |
| sll1800 | -1.346 | 0.000 | -1.324 | 0.000 | 50S ribosomal protein L4 |

| | | | | | |
|---|---|---|---|---|---|
| sll1078 | -1.348 | 0.000 | -1.443 | 0.000 | hydrogenase nickel incorporation protein HypA |
| slr6043 | -1.354 | 0.000 | -1.193 | 0.000 | probable cation efflux system protein, czcA homolog |
| sll1250 | -1.356 | 0.000 | -0.884 | 0.007 | hypothetical protein |
| slr1971 | -1.356 | 0.000 | -1.199 | 0.000 | hypothetical protein |
| slr1507 | -1.356 | 0.000 | -1.379 | 0.000 | hypothetical protein |
| ssr2611 | -1.359 | 0.000 | -1.293 | 0.000 | hypothetical protein |
| sll1841 | -1.360 | 0.000 | -1.232 | 0.000 | pyruvate dehydrogenase dihydrolipoamide acetyltransferase component (E2) |
| sll1951 | -1.361 | 0.000 | -1.268 | 0.000 | hemolysin |
| sll1814 | -1.365 | 0.000 | -1.322 | 0.000 | preprotein translocase SecY subunit |
| slr1203 | -1.370 | 0.000 | -1.098 | 0.000 | hypothetical protein |
| slr0895 | -1.370 | 0.000 | -1.400 | 0.000 | transcriptional regulator |
| slr1986 | -1.375 | 0.000 | -1.275 | 0.000 | allophycocyanin beta subunit |
| sll0996 | -1.375 | 0.000 | -1.284 | 0.000 | hypothetical protein |
| slr2076 | -1.377 | 0.000 | -1.296 | 0.000 | molecular chaperone GroEL |
| sll0749 | -1.381 | 0.000 | -1.207 | 0.000 | hypothetical protein |
| slr1876 | -1.384 | 0.000 | -1.408 | 0.000 | hypothetical protein |
| sll1097 | -1.386 | 0.000 | -1.315 | 0.000 | 30S ribosomal protein S7 |
| ssr1407 | -1.389 | 0.000 | -1.101 | 0.007 | hypothetical protein |
| sll0188 | -1.391 | 0.000 | -1.339 | 0.000 | unknown protein |
| sll0783 | -1.398 | 0.000 | -1.316 | 0.000 | unknown protein |
| sll0179 | -1.401 | 0.000 | -1.247 | 0.000 | glutamyl-tRNA synthetase |
| slr0204 | -1.409 | 0.000 | -1.257 | 0.000 | hypothetical protein YCF83 |
| slr0357 | -1.413 | 0.000 | -1.363 | 0.000 | histidyl-tRNA synthetase |
| sll8009 | -1.415 | 0.000 | -1.353 | 0.000 | type I restriction-modification system, M subunit |

| | | | | | |
|---|---|---|---|---|---|
| ssl3437 | -1.422 | 0.000 | -1.467 | 0.000 | 30S ribosomal protein S17 |
| slr1704 | -1.437 | 0.000 | -1.316 | 0.000 | hypothetical protein |
| sll1801 | -1.437 | 0.000 | -1.462 | 0.000 | 50S ribosomal protein L23 |
| slr1164 | -1.441 | 0.000 | -1.370 | 0.000 | ribonucleotide reductase subunit alpha |
| slr1367 | -1.443 | 0.000 | -1.290 | 0.000 | glycogen phosphorylase |
| ssl3436 | -1.445 | 0.000 | -1.461 | 0.000 | 50S ribosomal protein L29 |
| ssl1784 | -1.446 | 0.000 | -1.306 | 0.000 | 30S ribosomal protein S15 |
| slr1896 | -1.447 | 0.000 | -1.591 | 0.000 | hypothetical protein |
| slr6009 | -1.450 | 0.021 | -2.016 | 0.001 | unknown protein |
| slr6068 | -1.450 | 0.021 | -2.016 | 0.001 | unknown protein |
| sll1017 | -1.453 | 0.000 | -1.517 | 0.000 | ammonium transporter |
| ssl0453 | -1.453 | 0.000 | -1.358 | 0.000 | phycobilisome degradation protein NblA |
| slr1259 | -1.458 | 0.000 | -1.289 | 0.000 | hypothetical protein |
| smr0011 | -1.475 | 0.000 | -1.453 | 0.000 | 50S ribosomal protein L34 |
| sll1802 | -1.490 | 0.000 | -1.499 | 0.000 | 50S ribosomal protein L2 |
| sll0361 | -1.496 | 0.000 | -1.097 | 0.000 | hypothetical protein |
| slr1911 | -1.507 | 0.000 | -1.420 | 0.000 | hypothetical protein |
| slr0896 | -1.522 | 0.000 | -1.564 | 0.000 | multi-drug efflux transporter |
| ssr1398 | -1.524 | 0.000 | -1.420 | 0.000 | 50S ribosomal protein L33 |
| ssl2507 | -1.526 | 0.000 | -1.424 | 0.000 | unknown protein |
| sll1722 | -1.530 | 0.000 | -1.600 | 0.000 | glycosyl transferase |
| sll1805 | -1.538 | 0.000 | -1.588 | 0.000 | 50S ribosomal protein L16 |
| ssr1375 | -1.547 | 0.000 | -1.335 | 0.000 | hypothetical protein |
| sll0062 | -1.549 | 0.000 | -1.425 | 0.000 | hypothetical protein |
| sll0335 | -1.577 | 0.000 | -1.559 | 0.000 | hypothetical protein |

| | | | | | |
|---|---|---|---|---|---|
| slr6008 | -1.583 | 0.000 | -1.258 | 0.003 | unknown protein |
| slr6067 | -1.583 | 0.000 | -1.258 | 0.003 | unknown protein |
| sll0372 | -1.585 | 0.000 | -1.610 | 0.000 | hypothetical protein |
| slr6040 | -1.586 | 0.000 | -1.547 | 0.000 | two-component response regulator |
| sll0288 | -1.587 | 0.000 | -1.440 | 0.000 | septum site-determining protein MinC |
| sll0785 | -1.592 | 0.000 | -1.284 | 0.000 | unknown protein |
| sll1804 | -1.607 | 0.000 | -1.702 | 0.000 | 30S ribosomal protein S3 |
| sll0754 | -1.621 | 0.000 | -1.611 | 0.000 | ribosome binding factor A |
| ssr2016 | -1.625 | 0.000 | -1.093 | 0.006 | hypothetical protein |
| ssl3432 | -1.626 | 0.000 | -1.593 | 0.000 | 30S ribosomal protein S19 |
| sll1723 | -1.627 | 0.000 | -1.406 | 0.000 | colanic acid biosynthesis glycosyltransferase WcaL |
| sll1803 | -1.637 | 0.000 | -1.686 | 0.000 | 50S ribosomal protein L22 |
| sll1583 | -1.642 | 0.000 | -1.571 | 0.000 | unknown protein |
| ssl0452 | -1.662 | 0.000 | -1.667 | 0.000 | phycobilisome degradation protein NblA |
| sll1806 | -1.687 | 0.000 | -1.719 | 0.000 | 50S ribosomal protein L14 |
| slr1756 | -1.695 | 0.000 | -1.629 | 0.000 | glutamate--ammonia ligase |
| sll0172 | -1.701 | 0.000 | -1.436 | 0.000 | periplasmic protein, function unknown |
| sll1270 | -1.728 | 0.000 | -1.604 | 0.000 | glutamine-binding periplasmic protein/glutamine transport system permease protein |
| slr0882 | -1.735 | 0.000 | -1.544 | 0.000 | hypothetical protein YCF84 |
| slr0493 | -1.745 | 0.000 | -1.855 | 0.000 | similar to mannose-1-phosphate guanylyltransferase |
| slr1169 | -1.759 | 0.000 | -1.716 | 0.000 | unknown protein |
| slr1168 | -1.760 | 0.000 | -1.516 | 0.000 | unknown protein |
| sll0330 | -1.762 | 0.000 | -1.749 | 0.000 | 3-ketoacyl-ACP reductase |
| sll1813 | -1.796 | 0.000 | -1.872 | 0.000 | 50S ribosomal protein L15 |

| | | | | | |
|---|---|---|---|---|---|
| sll0786 | -1.806 | 0.000 | -1.387 | 0.000 | unknown protein |
| sll1745 | -1.812 | 0.000 | -1.856 | 0.000 | 50S ribosomal protein L10 |
| slr1912 | -1.821 | 0.000 | -1.630 | 0.000 | anti-sigma F factor antagonist |
| slr0288 | -1.827 | 0.000 | -1.773 | 0.000 | glutamine synthetase |
| sll0784 | -1.838 | 0.000 | -2.083 | 0.000 | nitrilase |
| sll0108 | -1.865 | 0.000 | -1.836 | 0.000 | Putative ammonium transporter |
| sll1812 | -1.871 | 0.000 | -1.874 | 0.000 | 30S ribosomal protein S5 |
| sll1811 | -1.900 | 0.000 | -1.950 | 0.000 | 50S ribosomal protein L18 |
| slr1452 | -1.905 | 0.000 | -1.977 | 0.000 | sulfate transport system substrate-binding protein |
| slr0364 | -1.939 | 0.000 | -1.759 | 0.000 | hypothetical protein |
| sll0622 | -1.946 | 0.000 | -1.839 | 0.000 | quinolinate synthetase |
| ssr1251 | -1.961 | 0.000 | -1.992 | 0.000 | hypothetical protein |
| sll0702 | -1.970 | 0.000 | -1.666 | 0.000 | unknown protein |
| sll7031 | -1.974 | 0.000 | -1.735 | 0.000 | hypothetical protein |
| sll0787 | -1.997 | 0.000 | -1.621 | 0.001 | hypothetical protein |
| sll1807 | -2.009 | 0.000 | -1.977 | 0.000 | 50S ribosomal protein L24 |
| ssr3465 | -2.012 | 0.000 | -1.438 | 0.000 | hypothetical protein |
| sll0789 | -2.022 | 0.000 | -1.866 | 0.000 | two-component response regulator OmpR subfamily |
| slr1726 | -2.079 | 0.000 | -1.711 | 0.000 | unknown protein |
| slr1167 | -2.109 | 0.000 | -2.097 | 0.000 | glycerol dehydrogenase |
| slr6096 | -2.114 | 0.035 | -3.215 | 0.001 | type I restriction-modification system, M subunit (fragment) |
| sll1808 | -2.147 | 0.000 | -2.059 | 0.000 | 50S ribosomal protein L5 |
| sll1810 | -2.171 | 0.000 | -2.130 | 0.000 | 50S ribosomal protein L6 |
| slr6039 | -2.180 | 0.000 | -2.337 | 0.000 | hypothetical protein |
| sll1809 | -2.186 | 0.000 | -2.182 | 0.000 | 30S ribosomal protein S8 |

| | | | | | |
|---|---|---|---|---|---|
| slr0750 | -2.335 | 0.000 | -2.390 | 0.000 | light-independent protochlorophyllide reductase subunit N |
| sll0788 | -2.382 | 0.000 | -2.517 | 0.000 | hypothetical protein |
| sll1688 | -2.434 | 0.000 | -2.312 | 0.000 | threonine synthase |
| slr1841 | -2.440 | 0.000 | -2.373 | 0.000 | membrane protein |
| slr6042 | -2.452 | 0.000 | -2.389 | 0.000 | probable cation efflux system protein, czcB homolog |
| sll1271 | -2.486 | 0.000 | -2.550 | 0.000 | hypothetical protein |
| ssr1399 | -2.522 | 0.000 | -2.316 | 0.000 | 30S ribosomal protein S18 |
| slr0749 | -2.938 | 0.000 | -2.754 | 0.000 | protochlorophyllide reductase iron-sulfurATP-binding protein |
| slr0447 | -3.074 | 0.000 | -3.060 | 0.000 | urea ABC transporter substrate-binding protein |
| slr2135 | -3.296 | 0.000 | -3.387 | 0.000 | hydantoin utilization protein A |
| ssr6019 | -3.410 | 0.005 | -2.315 | 0.027 | unknown protein |
| ssr6078 | -3.410 | 0.005 | -2.315 | 0.027 | unknown protein |
| slr0946 | -3.422 | 0.000 | -3.405 | 0.000 | arsenate reductase |
| slr0793 | -3.807 | 0.000 | -3.636 | 0.000 | cation efflux system protein involved in nickel and cobalt tolerance |
| slr7076 | -4.588 | 0.002 | -4.073 | 0.002 | hypothetical protein |
| slr0945 | -4.854 | 0.000 | -4.993 | 0.000 | NADPH-dependent quinone reductase ArsH |
| slr0944 | -6.096 | 0.000 | -6.397 | 0.000 | transporter |
