# Supplementary material for: Two types of C-terminal regions of RNA-binding proteins play distinct roles in stress tolerance of Synechocystis sp. PCC 6803
Source: FEMS Microbiol Lett. 2022 Feb 25;369(1):fnac021. doi: 10.1093/femsle/fnac021 (PMC9333190; doi:10.1093/femsle/fnac021)
Supplement: fnac021_Supplemental_Files [file fnac021_supplemental_files.zip › Supplemental_figures.pdf]

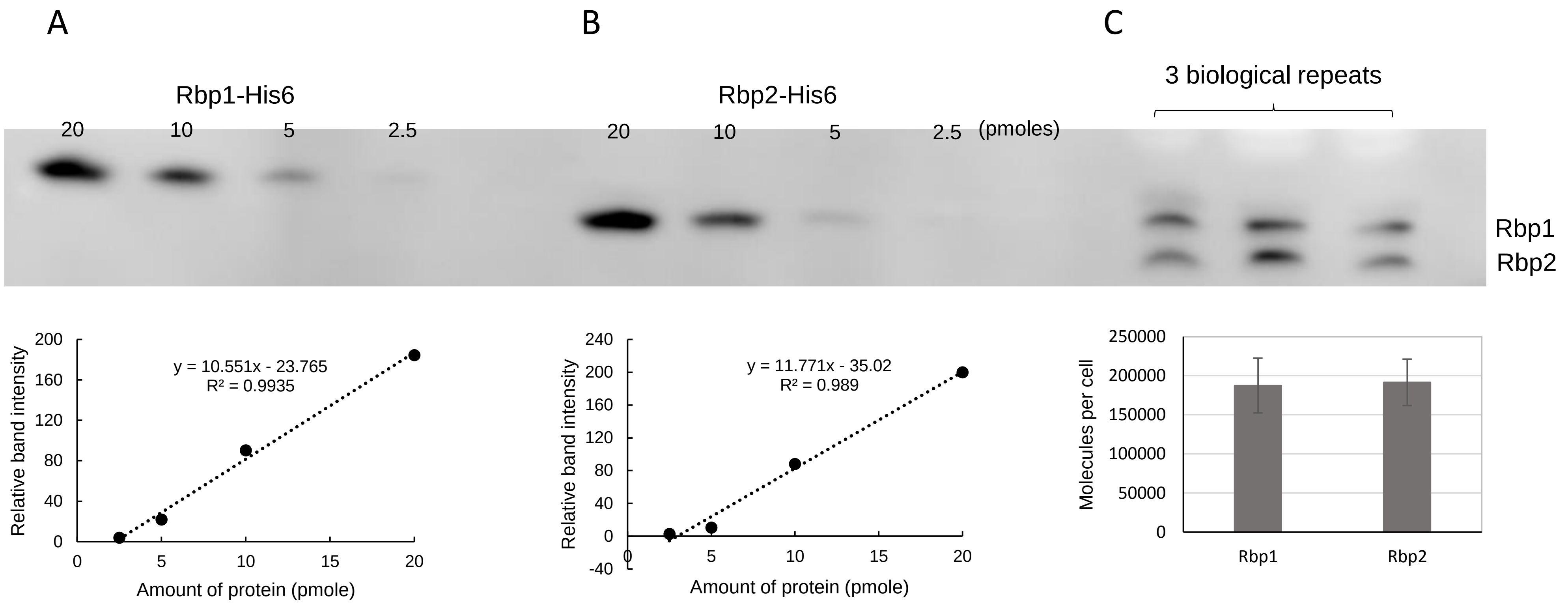

Fig. S1 Quantification of Rbp1 and Rbp2 in *Synechocystis* 6803 exposed to 15 °C for 48 h. (A) Linear relationship (lower) between the amount of recombinant Rbp1 and the relative band intensity on Western blot (upper); (B) Linear relationship (lower) between the amount of recombinant Rbp2 and the relative band intensity on Western blot (upper); (C) Evaluation of cellular concentrations of Rbp1 and Rbp2 in *Synechocystis* 6803 (lower) based on band intensities on the Western blot (upper, 3 biological repeats). It is worth mentioning that the antiserum used in this study was against the recombinant Rbp2, whereas that used in the previous publication (J Bacterial, 2011, 193: 2675-2683) was against the recombinant Rbp1.

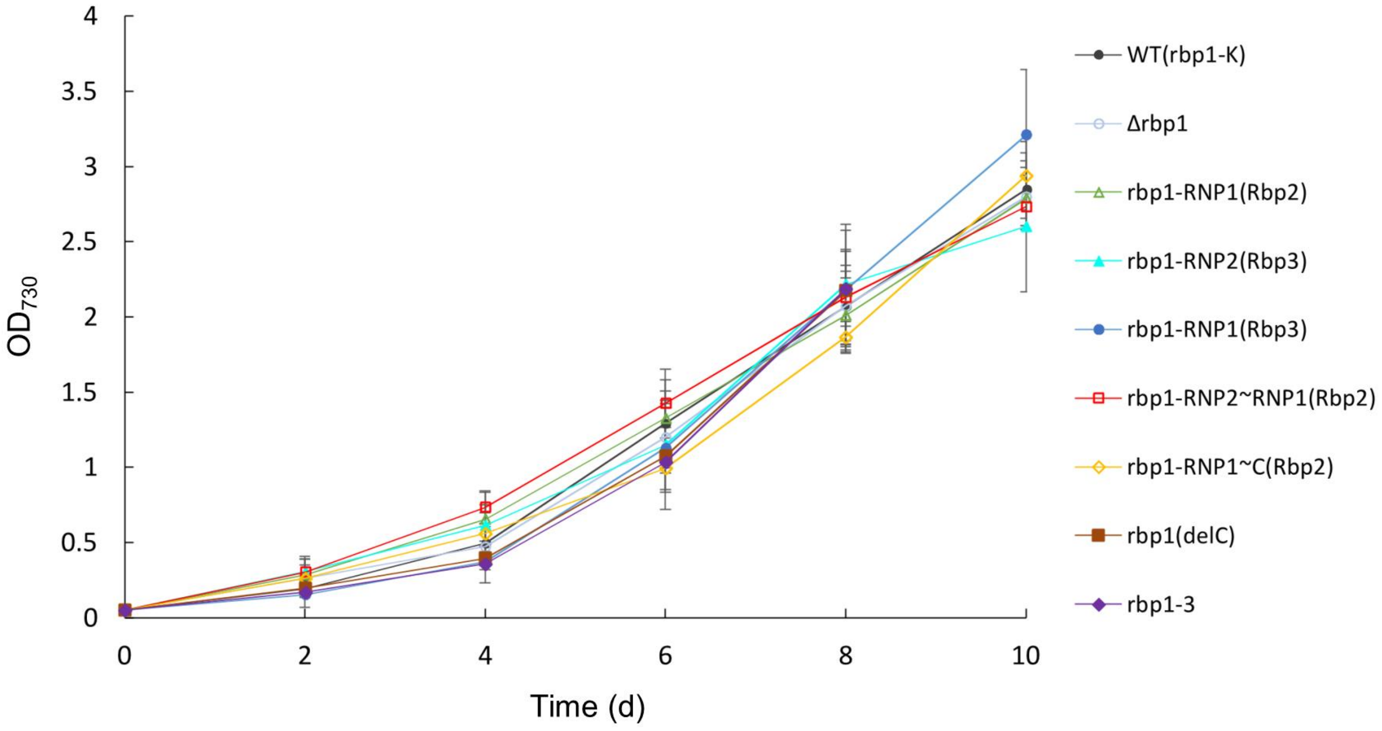

Fig. S2 Growth of the WT (*rbp1*-K) strain and *rbp1* mutants of *Synechocystis* 6803 in BG11 at 30 ^{o}C. *rbp1-3* here refers to the *rbp1-3*a strain.

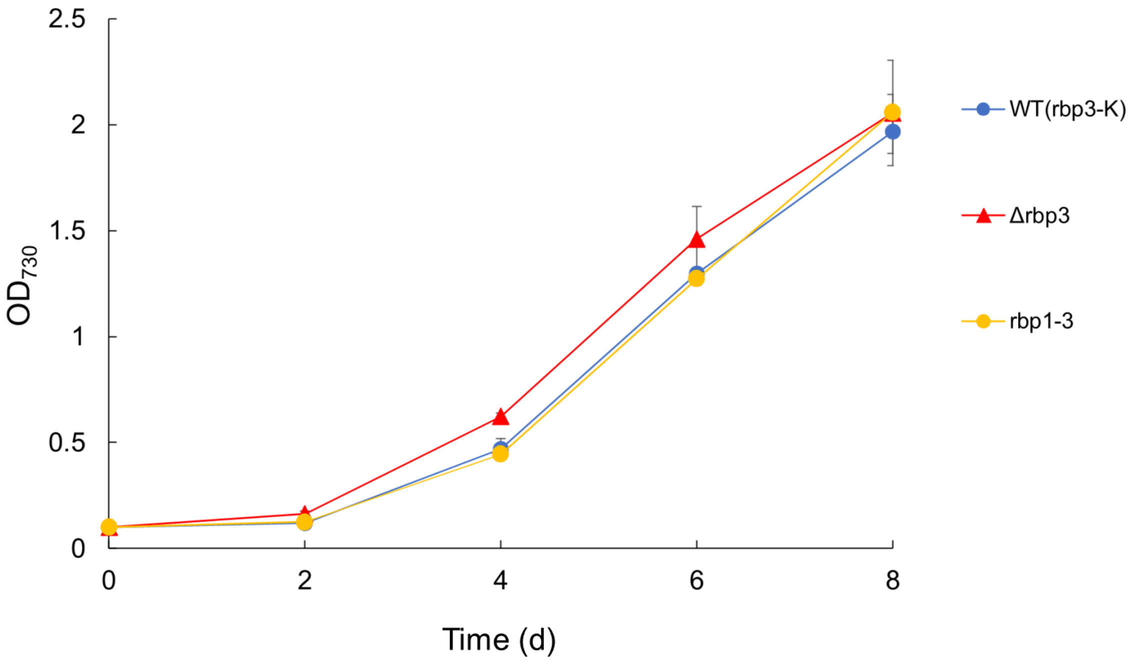

Fig. S3 Growth of the WT (*rbp3*-K) strain and *rbp3* mutants of *Synechocystis* 6803 in BG11 at 30 °C. *rbp1-3* here refers to the *rbp1-3*b strain.

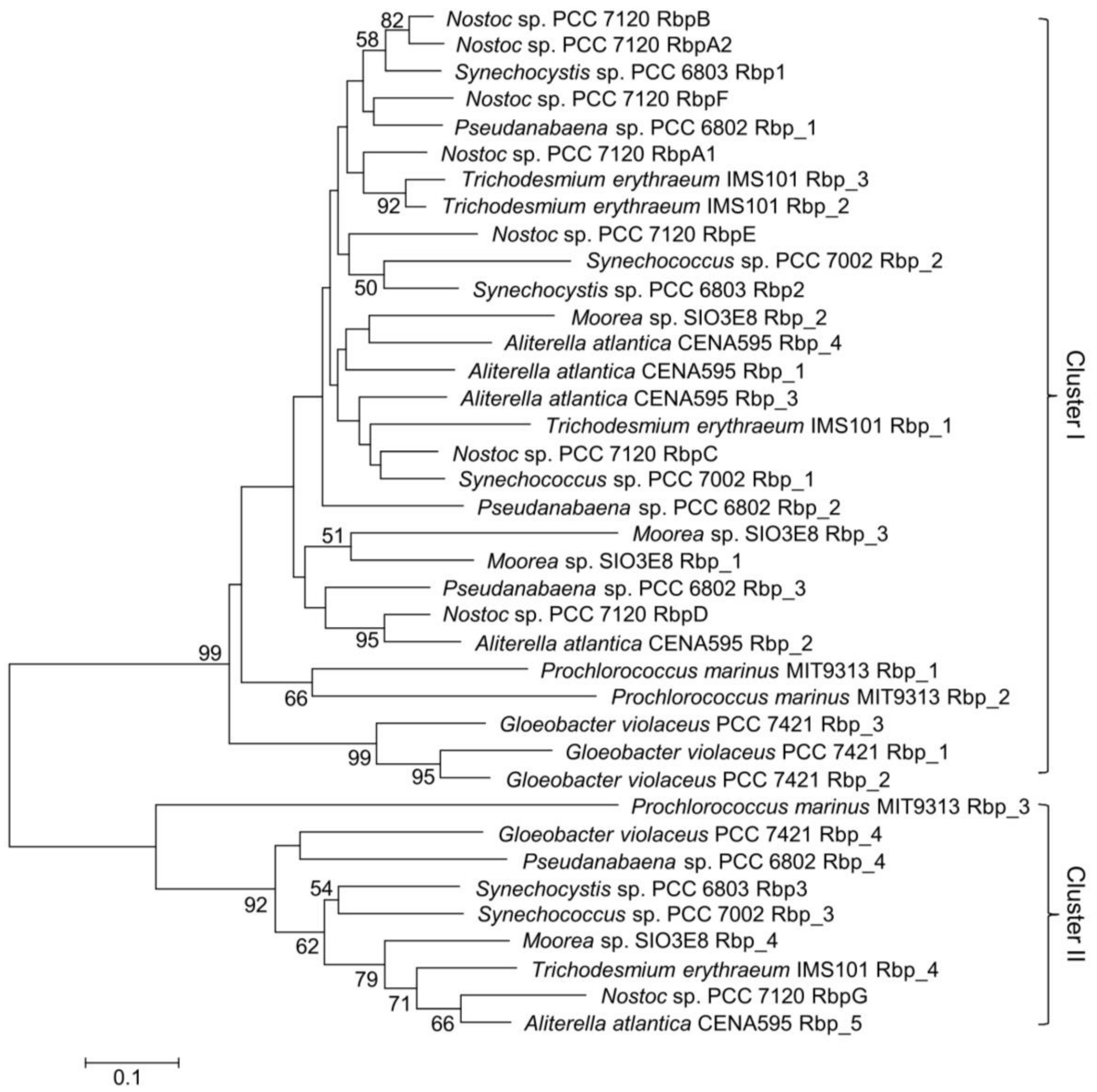

Fig. S4 Phylogenetic tree of RNA-binding proteins. The tree was constructed by MEGA 6 using the Neighbor-Joining method with 1000 bootstrap replicates. Bootstrap values (≥ 50) are shown beside branches. The scale bar shows expected substitutions per site.
