# Supplementary material for: Two types of C-terminal regions of RNA-binding proteins play distinct roles in stress tolerance of Synechocystis sp. PCC 6803
Source: FEMS Microbiol Lett. 2022 Feb 25;369(1):fnac021. doi: 10.1093/femsle/fnac021 (PMC9333190; doi:10.1093/femsle/fnac021)
Supplement: fnac021_Supplemental_Files [file fnac021_supplemental_files.zip › Table_S1.pdf]

Table S1 Cyanobacterial strains, plasmids and primers

Strain, plasmid, or primer	Derivation, relevant characteristics, or sequences (5′→3′) [a]	Source or reference(s)
Synechocystis 6803 strains [b]		
Synechocystis sp. PCC 6803	Wild type (WT)	Zhao J, Peiking University
DRHB2548	(Cm^r)Em^r, *rbp1*::C.CE2, with *Synechocystis* sp. chromosomal (NCBI GenBank accession no. BA000022.2) bp 3181992-3181754 replaced by C.CE2, a Cm^rEm^r cassette	Tan et al. 2011
DRHB5509	Sp^r, Δ*rbp3*, with *rbp3* (*Synechocystis* sp. chromosomal bp 2724627-2725103) replaced by Ω, a Sp^r cassette	This study
DRHB5511	Km^r, *rbp1-3*b, with the RRM region of *rbp3* (*Synechocystis* sp. chromosomal bp 2724648-2724899) replaced with the counterpart of *rbp1* (*Synechocystis* sp. chromosomal bp 3182013-3181771), and the C.K2 cassette inserted immediately downstream of *rbp1-3*	This study
DRHB5899	Km^r, WT (*rbp3*-K), with the C.K2 cassette inserted immediately downstream of *rbp3*	This study
DRHB5971	Km^r, Δ*rbp1*, with *rbp1* (*Synechocystis* sp. chromosomal bp 3182236-3181708) replaced by C.K2	This study
DRHB6001	Km^r, *rbp1*(delC), with the C-terminal region of *rbp1* (*Synechocystis* sp. chromosomal bp 3181770-3181708) removed, and the C.K2 cassette inserted immediately downsteam of *rbp1*(delC)	This study
DRHB6003	Km^r, *rbp1-3*a, with the C-terminal region of *rbp1* (*Synechocystis* sp. chromosomal bp 3181770-3181708) replaced with the longer C-terminal region of *rbp3* (*Synechocystis* sp. chromosomal bp 2724900-2725103), and the C.K2 cassette inserted immediately downstream of *rbp1-3*	This study
DRHB6005	Km^r, *rbp1*-RNP1(Rbp2), with CTA at *Synechocystis* sp. chromosomal bp 3181869-3181867	This study

	substituted with ATG, replacing the RNP1 of Rbp1 with that of Rbp2, and the C.K2 cassette inserted immediately downstream of *rbp1*-RNP1(Rbp2)	
DRHB6006	Km^r, *rbp1*-RNP2(Rbp3), with CGTCTC inserted between chromosomal bp 3182005-3182004, generating a Rbp3 RNP2 encoding sequence in *rbp1*, and the C.K2 inserted immediately downstream of *rbp1*-RNP2(Rbp3)	This study
DRHB6007	Km^r, *rbp1*-RNP1(Rbp3), with GGTTTTGTCGAGCTA (*Synechocystis* sp. chromosomal bp 3181881-3181867) substituted with GCTTTTGTAACGGTT, replacing RNP1 of Rbp1 with that of Rbp3, and the C.K2 cassette inserted immediately downstream of *rbp1*-RNP1(Rbp3)	This study
DRHB6008	Km^r, *rbp1*-RNP2~RNP1(Rbp2), with the portion between RNP2 and RNP1 of *rbp1* (*Synechocystis* sp. chromosomal bp 3181986-3181936) replaced with the counterpart of *rbp2* (*Synechocystis* sp. chromosomal bp 1135364-1135414), and the C.K2 cassette inserted immediately downstream of *rbp1*-RNP2~RNP1(Rbp2)	This study
DRHB6009	Km^r, *rbp1*-RNP1~C(Rbp2) with the portion between RNP1 and C-terminal region of *rbp1* (*Synechocystis* sp. chromosomal bp 3181866-3181774) replaced with the counterpart of *rbp2* (*Synechocystis* sp. chromosomal bp 1135484-1135576), and the C.K2 inserted immediately downstream of *rbp1*-RNP2~RNP1(Rbp2)	This study
DRHB6010	Km^r, WT (*rbp1*-K), with the C.K2 cassette inserted immediately downstream of *rbp1*	This study
Plasmids [c]		
pET21b	Ap^r, expression vector	Milipore
pHB576	Cm^rSp^r, a cloning vector	Dr. Gao Hong, Institute of Hydrobiology, CAS
pHB5501	Ap^r, the PCR fragment upstream of *rbp3* (*Synechocystis* sp. chromosomal bp 2723674-2724626), amplified with primer pairs $P_{slr0193}$-1/-2, cloned into pMD18-T	This study

pHB5502	Ap^r, the PCR fragment downstream of *rbp3* (*Synechocystis* sp. chromosomal 2725104-2725915), amplified with primer pairs $P_{slr0193}$-3/-4, cloned into pMD18-T	This study
pHB5503	Ap^r, the PCR fragment containing *rbp1-3*, with the glycine-rich C-terminal region of *rbp1* (*Synechocystis* sp. chromosomal bp. 3181770-3181708) replaced with the long C-terminal region from *rbp3* (*Synechocystis* sp. chromosomal 2724900-2725103), generated by overlap PCR (Horton et al, 1989) using primer pairs *rbp1*-N-1/*rbp1-3*-R and *rbp3*-C-2/*rbp1-3*-F, cloned into pMD18-T	This study
pHB5505	Ap^r Sp^r, the Ω cassette excised with BamH I from pHB576, cloned into BamH I-cut pHB5502	This study
pHB5506	Ap^r Km^r, the C.K2 cassette excised with BamH I from pRL446 and blunted with T4 DNA polymerase, cloned into Xho I-cut and T4 DNA polymerase-blunted pHB5503	This study
pHB5509	Ap^r Sp^r, the DNA fragment containing the Sp^r cassette and the sequence downstream of *rbp3* excised with Pvu II and Sca I from pHB5505, cloned into Sal I-cut and T4 DNA polymerase-blunted pHB5501, forming the upstream-Sp^r-downstream structure for generating the Δ*rbp3* mutant of *Synechocystis* 6803	This study
pHB5510	Ap^r Km^r, the *rbp1-3*-Km^r fragment excised with Pvu II from pHB5506, cloned into Sal I-cut and T4 DNA polymerase-blunted pHB5501	This study
pHB5511	Ap^r Km^r, the upstream-*rbp1-3*-Km^r fragment excised with Pvu II from pHB5510, cloned into BamH I-cut and and T4 DNA polymerase-blunted pHB5502	This study
pHB5743	Ap^r, the PCR fragment containing *rbp1*-RNP1(Rbp2), with RNP1 of Rbp1 replaced with that of Rbp2, generated by overlap PCR using primer pairs *rbp1*-F/RNP1(1>2)-1 and RNP1(1>2)-2/*rbp1*-R, cloned into pMD18-T	This study
pHB5744	Ap^r, the PCR fragment containing *rbp1*-RNP1(Rbp3), with RNP1 of Rbp1 replaced with that of Rbp3, generated by overlap PCR using primer pairs *rbp1*-F/RNP1(1>3)-1 and RNP1(1>3)-2/*rbp1*-R, cloned into pMD18-T	This study

pHB5745	Ap^r, the PCR fragment containing *rbp1*-RNP2(Rbp3), with RNP2 of Rbp1 replaced with that of Rbp3, amplified with primer pairs RNP2(1>3) and *rbp1*-R, cloned into pMD18-T	This study
pHB5746	Ap^r, the PCR fragment containing *rbp1*-RNP1~C(Rbp2), with the portion between RNP1 and the C-terminal region of Rbp1 (encoded by *Synechocystis* sp. chromosomal bp 3181866-3181774) replaced with the counterpart of Rbp2 (encoded by *Synechocystis* sp. chromosomal bp 1135484-1135576), generated by overlap PCR using primer pairs *rbp1*-F/RNP1~C(1>2)-1, RNP1~C(1>2)-2/RNP1~C(1>2)-3 and RNP1~C(1>2)-4/*rbp1*-R, cloned into pMD18-T	This study
pHB5747	Ap^r, the PCR fragment containing *rbp1*-RNP2~RNP1(Rbp2),with the portion between RNP2 and RNP1 of Rbp1 (encoded by *Synechocystis* sp. chromosomal bp 3181986-3181885) replaced with the counterpart of Rbp2 (encoded by *Synechocystis* sp. chromosomal bp 1135364-1135465), amplified with primer pairs RNP2~RNP1(1>2) / *rbp1*-R, cloned into pMD18-T	This study
pHB5848	Ap^r, the PCR fragment containing the sequence of *rbp3* (*Synechocystis* sp. chromosomal 2724627-2725106), amplified with primers *rbp3*-N-1/*rbp3*-C-2, cloned into pMD18-T	This study
pHB5861	Ap^r Km^r, the C.K2 cassette excised with BamH I from pRL446 (NCBI GenBank accession no. EU346690) and blunted with T4 DNA polymerase, cloned into Sal I-cut and T4 DNA polymerase-blunted pHB5848	This study
pHB5886	Ap^r Km^r, the *rbp3*-C.K2 fragment excised with Kpn I and Sph I from pHB5848, blunted with T4 DNA polymerase, cloned into Sal I-cut and T4 DNA polymerase-blunted pHB5501	This study
pHB5899	Ap^r Km^r, the upstream-*rbp3*-C.K2 fragment excised with Pvu II and Sca I, cloned into Xba I-cut and T4 DNA polymerase-blunted pHB5502	This study

pHB5970	Ap^r, the PCR fragment containing sequences upstream (*Synechocystis* sp. chromosomal bp 3183174-3182237) and downstream (*Synechocystis* sp. chromosomal bp 3181707-3180526) of the *rbp1* encoding region, generated by overlap PCR using primer pairs P_{rbp1}-1/-2 and P_{rbp1}-3/-4, excised with Sac I and Pst I, cloned into Sac I- and Pst I-cut pMD18-T	This study
pHB5971	Ap^r Km^r, the C.K2 cassette excised with BamH I from pRL446, blunted with T4 DNA polymerase, cloned into Xho I-cut and T4 DNA polymerase-blunted pHB5970	This study
pHB5972	Ap^r, the PCR fragment containing the promotor of *rbp1* (*Synechocystis* sp. chromosomal bp 3182242-3182021) and *rbp1*(delC), amplified with primer pairs *rbp1*-T-1/*rbp1*-delC-4, cloned into pMD18-T	This study
pHB5974	Ap^r, the PCR fragment containing the promotor of *rbp1* and *rbp1-3*, generated by overlap PCR using primer pairs *rbp1*-T-1/-2 and *rbp1*-T-3/*rbp1-3*-4, with *Synechocystis* 6803 DNA and pHB5506 as the templates respectively, cloned into pMD18-T	This study
pHB5976	Ap^r, the PCR fragment containing the promotor of *rbp1* and *rbp1*-RNP1(Rbp2), generated by overlap PCR using primer pairs *rbp1*-T-1/-2 and *rbp1*-T-3/-4, with *Synechocystis* 6803 DNA and pHB5743 as the templates respectively, cloned into pMD18-T	This study
pHB5977	Ap^r, the PCR fragment containing the promotor of *rbp1* and *rbp1*-RNP2(Rbp3), generated by overlap PCR using primer pairs *rbp1*-T-1/-2 and *rbp1*-T-3/-4, with *Synechocystis* 6803 DNA and pHB5745 as the templates respectively, cloned into pMD18-T	This study
pHB5978	Ap^r, the PCR fragment containing the promotor of *rbp1* and *rbp1*-RNP1(Rbp3), generated by overlap PCR using primer pairs *rbp1*-T-1/-2 and *rbp1*-T-3/-4, with *Synechocystis* 6803 DNA and pHB5744 as the templates respectively, cloned into pMD18-T	This study

pHB5979	Ap^r, the PCR fragment containing the promotor of *rbp1* and *rbp1*-RNP2~RNP1(Rbp2), generated by overlap PCR using primer pairs *rbp1*-T-1/-2, and *rbp1*-T-3/-4, with *Synechocystis* 6803 DNA and pHB5747 as the templates respectively, cloned into pMD18-T	This study
pHB5980	Ap^r, the PCR fragment containing the promotor of *rbp1* and *rbp1*-RNP1~C(Rbp2), generated by overlap PCR using primer pairs *rbp1*-T-1/-2 and *rbp1*-T-3/-4, with *Synechocystis* 6803 DNA and pHB5746 as the templates respectively, cloned into pMD18-T	This study
pHB5981	Ap^r, the PCR fragment containing the promotor and the encoding region of *rbp1*, amplified with primer pairs *rbp1*-T-1/-4, cloned into pMD18-T	This study
pHB6001	Ap^r Km^r, *rbp1*(delC) excised with Apa I and Kpn I from pHB5972, cloned into Apa I- and Kpn I-cut pHB5971	This study
pHB6003	Ap^r Km^r, *rbp1-3* excised with Apa I and Kpn I from pHB5974, cloned into Apa I- and Kpn I-cut pHB5971	This study
pHB6005	Ap^r Km^r, *rbp1*-RNP1(Rbp2) excised with Apa I and Kpn I from pHB5976, cloned into Apa I- and Kpn I-cut pHB5971	This study
pHB6006	Ap^r Km^r, *rbp1*-RNP2(Rbp3) excised with Apa I and Kpn I from pHB5977, cloned into Apa I- and Kpn I-cut pHB5971	This study
pHB6007	Ap^r Km^r, *rbp1*-RNP1(Rbp3) excised with Apa I and Kpn I from pHB5978, cloned into Apa I- and Kpn I-cut pHB5971	This study
pHB6008	Ap^r Km^r, *rbp1*-RNP2~RNP1(Rbp2) excised with Apa I and Kpn I from pHB5979, cloned into Apa I- and Kpn I-cut pHB5971	This study
pHB6009	Ap^r Km^r, *rbp1*-RNP1~C(Rbp2) excised with Apa I and Kpn I from pHB5980, cloned into Apa I- and Kpn I-cut pHB5971	This study
pHB6010	Ap^r Km^r, *rbp1* excised with Apa I and Kpn I from pHB5981, cloned into Apa I and Kpn I-cut pHB5971	This study

pHB6596	Ap^r, PCR fragment containing *rbp1*(delC), amplified with primer pairs Rbp1-K0/Rbp1(delC)R-Xho I, excised with Nde I and Xho I, cloned into Nde I- and Xho I-cut pET-21b	This study
pHB7083	Ap^r, PCR fragment containing *rbp1*, amplified with primer pairs Rbp1-K0/-K2, excised with Nde I and Xho I, cloned into Nde I- and Xho I-cut pET21b	This study
pHB7084	Ap^r, PCR fragment containing *rbp2*, amplified with primer pairs Rbp2-K0/-K2, excised with Nde I and Xho I, cloned into Nde I- and Xho I-cut pET21b	This study
pMD18-T	Ap^r, cloning T-vector	Takara
pRL446	Ap^r Km^r, a plasmid with the C.K2 cassette	Elhai J and Wolk CP, 1988
Primers (5′→3′)		
rbp1-F	CTCAGTTTTTGGAGAAAATCCATGTC	
rbp1-R	CTAGTAGCGGCTACCACCATAG	
RNP1(1>2)-1	GGAGAAAATCCATGTCCATTTATGTCGGGAACC	
RNP1(1>2)-2	CGGTTTTGTCGAGATGGAAGCTGACGCCGAAGAAACG	
RNP1(1>3)-1	CAGCTTCAACCGTTACAAAAGCGAAGCCCCGCATGCGAC	
RNP1(1>3)-2	CTTCGCTTTTGTAACGGTTGAAGCTGACGCCGAAGAAAC	
RNP2(1>3)	CTCAGTTTTTGGAGAAAATCCATGTCAATTCGTCTCTATGTAGGCAACCTGTCCTATGA	
RNP1~C(1>2)-1	CTTCTTCCTTATCGGAAGATAGCTCGACAAAACCGAAGC	
RNP1~C(1>2)-2	CGGTTTTGTCGAGCTATCTTCCGATAAGGAAGAAGATGC	
RNP1~C(1>2)-3	CCGCCACTGCGATTTTCTCTCGGTCTTGCTTTATTAAC	
RNP1~C(1>2)-4	GTTAATAAAGCAAGACCGAGAGAAAATCGCAGTGGCGG	
RNP2~RNP1(1>2)	CTCAGTTTTTGGAGAAAATCCATGTCAATTTATGTAGGCAACCTGTCCTACCAAGCCACCGAAGATGACGTTTTGACTGTCTTCTCCGAGTATGGCACTGTAAAGCGGGTTCAGCTC	
P_{rbp1}-1	CCTGAGCTCTCGTAGCCCAAAGCCACCATT	

P*rbp1*-2	CTAAGCCCTCGAGGGGCCCTCCGGTACCATTGCTTAGACGGCTCG
P*rbp1*-3	AGCAATGGTACCGGAGGGCCCCTCGAGGGCTTAGTTTTTGTTCGCCGTC
P*rbp1*-4	AACTGCAGCCGATTCAACTACCGCTTCTGTTG
rbp1-T-1	GGTACCATTCTTTTCTGGTTAC
rbp1-T-2	CTCCAAAAACTGAGGGATATGTAGAGGGTGAT
rbp1-T-3	ACATATCCCTCAGTTTTTGGAGAAAATCCATGTC
rbp1-T-4	GGGCCCCTAGTAGCGGCTACCACCAT
rbp1-delC-4	GGGCCCCTATTCCCGGGGCTTGGC
P*slr0193*-1	CGGACTTCACCACTATTTACGA
P*slr0193*-2	ATTTTCTGTAGGAGAAATTGCTCGGGC
P*slr0193*-3	GTCCACAGGTTTTCCTGAACC
P*slr0193*-4	AACTCCGCTGGGTTGGTAA
rbp1-N-1	CCCTCAGTTTTTGGAGAAAATCCATGT
rbp1-3-F	AACAAAGCCAAGCCCCGGGAAAAGGATGACGAAGAAGGGGGCA
rbp1-3-R	TGCCCCCTTCTTCGTCATCCTTTTCCCGGGGCTTGGCTTTGTT
rbp3-C-2	GACCTACTGGGCCGCTGTCA
rbp3-N-1	ATCCCCGGAGGTATTTAGATCATGT
Rbp1-K0	GGATCCCATATGTCAATTTATGTAGGCAACCTGTCC
Rbp1-K2	ATCTCGAGGTAGCGGCTACCACCA
Rbp2-K0	GGATCCCATATGTCCATTTATGTCGGGAACC
Rbp2-K2	ATCTCGAGACGAGGGGTTCTCGGTC
Rbp1(delC)R-XhoI	CCCTCGAGTTCCCGGGGCTTGGCTTTG

[a] Ap, ampicillin; Cm, chloramphenicol; Em, erythromycin; Km, kanamycin; Sp, spectinomycin.

[b] DRHB(number) refers to a product of double homologous recombination between plasmid pHB(number) and the *Synechocystis* sp. genome. For example, DRHB5509 is the strain generated by transformation of pHB5509 into *Synechocystis* sp. PCC 6803.

[c] Unless stated otherwise, the template for PCRs was *Synechocystis* genomic DNA.

References

1. Elhai J, Wolk CP. 1988. A versatile class of positive-selection vectors based on the nonviability of palindrome-containing plasmids that allows cloning into long polylinkers. Gene 68: 119-138
2. Horton R, Hunt HD, Ho SN, Pullen JK, Pease LR. 1989. Engineering hybrid genes without the use of restriction enzymes: gene splicing by overlap extension. Gene 77: 61-68.
3. Tan X, Zhu T, Shen S, Yin C, Gao H, Xu X. 2011. Role of Rbp1 in the acquired chill-light tolerance of cyanobacteria. J Bacteriol 193: 2675-83.
