# Supplementary material for: Two types of C-terminal regions of RNA-binding proteins play distinct roles in stress tolerance of Synechocystis sp. PCC 6803
Source: FEMS Microbiol Lett. 2022 Feb 25;369(1):fnac021. doi: 10.1093/femsle/fnac021 (PMC9333190; doi:10.1093/femsle/fnac021)
Supplement: fnac021_Supplemental_Files [file fnac021_supplemental_files.zip › Table_S2.pdf]

Table S2 RNA-binding proteins used in the phylogenetic analysis

Species	GenBank assembly accession no.	ID/location	Name of Protein
Synechocystis sp. PCC 6803	GCA_000009725.1	sll0517*	Rbp1
		ssr1480*	Rbp2
		slr0193*	Rbp3
Nostoc / *Anabaena* sp. PCC 7120	GCA_000009705.1	alr0741*	RbpA1
		alr2087*	RbpA2
		all2928*	RbpB
		alr4683*	RbpC
		asl4022*	RbpD
		all2777*	RbpE
		alr2311*	RbpF
		all4377*	RbpG
Gloeobacter violaceus PCC 7421	GCA_000011385.1	gll1391*	Rbp_1
		gll2326*	Rbp_2
		gll2530*	Rbp_3
		glr2925*	Rbp_4
Aliterella atlantica CENA595	GCA_000952155.1	KJH70491.1^	Rbp_1
		KJH70591.1^	Rbp_2
		KJH72344.1^	Rbp_3
		KJH73506.1^	Rbp_4
		KJH73379.1^	Rbp_5
Moorea sp. SIO3E8	GCA_010692445.1	NEO11686.1^	Rbp_1
		NEO14469.1^	Rbp_2
		NEO15721.1^	Rbp_3
		NEO14892.1^	Rbp_4
Synechococcus sp. PCC 7002	GCA_000019485.1	ACA99619.1^	Rbp_1
		ACA98282.1^	Rbp_2
		ACA99265.1^	Rbp_3
Trichodesmium erythraeum IMS101	GCA_000014265.1	ABG51119.1^	Rbp_1
		ABG53665.1^	Rbp_2
		ABG53664.1^	Rbp_3
		ABG53028.1^	Rbp_4
Prochlorococcus marinus MIT9313	BX548175.1	CAE21256.1^	Rbp_1
		CAE20189.1^	Rbp_2
		CAE22176.1^	Rbp_3
Pseudanabaena sp. PCC 6802	GCA_000332175.1	270144..270452†	Rbp_1
		2777950..2778264†	Rbp_2
		1534072..1534359†	Rbp_3
		1009266..1009757†	Rbp_4

* ORF_ID; ^ Protein_ID; † genome location of ORF.
